# Is It Time to Start Worrying? A Comprehensive Report on the Three-Year Prevalence of ESBL-Producing Bacteria and Their Trends in Antibiotic Resistance from the Largest University Hospital in Slovakia

**DOI:** 10.3390/ph17111517

**Published:** 2024-11-11

**Authors:** Yashar Jalali, Andrea Kološová, Adriána Liptáková, Ján Kyselovič, Anna Oleárová, Monika Jalali, Juraj Payer

**Affiliations:** 1 Faculty of Medicine, Comenius University in Bratislava, 5th Department of Internal Medicine, University Hospital Bratislava, Ružinov, Špitálska 24, 813 72 Bratislava, Slovakia, and Ružinovská 6, 826 06 Bratislava, Slovakia; jan.kyselovic@fmed.uniba.sk (J.K.); adamcova.monika@gmail.com (M.J.); juraj.payer@fmed.uniba.sk (J.P.); 2 Department of Hospital Hygiene and Epidemiology, University Hospital Bratislava, Ružinov, Ružinovská 4810/6, 821 01 Bratislava, Slovakia; kolosova@ru.unb.sk; 3 Institute of Microbiology, Faculty of Medicine, Comenius University in Bratislava, Špitálska 24, 813 72 Bratislava, Slovakia; adriana.liptakova@fmed.uniba.sk; 4 Department of Clinical Pharmacology, University Hospital Bratislava, Ružinov, Ružinovská 4810/6, 821 01 Bratislava, Slovakia; olearova@ru.unb.sk

**Keywords:** ESBL, extended-spectrum beta-lactamase, antimicrobial resistance, *Escherichia coli*, *Klebsiella pneumoniae*, *Proteus mirabilis*

## Abstract

Background/Objectives: Over the past few decades, extended-spectrum β-lactamase (ESBL)-producing bacteria have become a great concern in healthcare systems worldwide, imposing large burdens by increasing antimicrobial resistance and patient morbidity. Given the high mortality rates and emergence of multidrug-resistant (MDR) strains, monitoring ESBL prevalence and resistance patterns is crucial. This study aimed to evaluate ESBL-producing *Escherichia coli*, *Proteus mirabilis*, and *Klebsiella pneumoniae* over three years, focusing on phenotypic distribution and resistance profiles. Methods: A total of 1599 ESBL-producing bacterial samples were collected and analysed. A panel of 20 antibiotics was tested to determine resistance traits. Data were recorded on phenotypical distribution, isolation types, changes in antibiotic resistance, and the relation of such changes to antibiotic consumption (defined daily dose) from clinical isolates. Results: Phenotypical analysis revealed the minimal presence of the Cefotaximase from Munich (CTX-M) phenotype in *E. coli* and *K. pneumoniae*, creating a distinct epidemiological profile compared to global patterns. Shifts in isolation trends, particularly in *P. mirabilis*, suggest an expected increase in associated-mortality-rate in the coming years. While resistance trends were not statistically significant, MDR and extensively drug-resistant (XDR) strains were identified across all three bacteria. Only meropenem showed consistent 100% efficacy against *E. coli*, with other antibiotics displaying only partial effectiveness. Conclusions: These findings highlight the need for ongoing surveillance of ESBL-producing bacteria and underscore challenges in managing antibiotic resistance due to limited efficacy of last-resort treatments. The unique phenotypical distribution observed could impact local resistance management strategies in hospital settings in the coming years.

## 1. Introduction

For nearly six decades, efforts to develop new antibiotics effective against Gram-negative bacteria have been unsuccessful [1]. During this period, Gram-negative bacteria have acquired resistance to most available antibiotics through different, rather sophisticated drug-resistance mechanisms [2]. The lack of options for the treatment of antibiotic-resistant Gram-negative bacteria has become a concerning challenge in modern clinical infectiology. Annually, nearly 33,000 people die from infections caused by drug-resistant bacteria in Europe [3]. Cassini et al. estimated that the burden of antimicrobial resistance in Europe is comparable to that of HIV, influenza, and tuberculosis combined [4]. By 2050, it is estimated that nearly 10 million lives per year will be at risk due to the rise in drug-resistant infections [5,6].

β-Lactamases are enzymes that provide bacterial resistance to antibiotics from the β-lactam family by hydrolysing the amid group of the fourth member of the beta-lactam chain [7,8]. β-Lactamases are coded by chromosomal and/or plasmid DNA and can be easily transferred among bacteria, rapidly spreading resistance traits among different bacteria [9]. Although these enzymes are produced by both Gram-positive and Gram-negative bacteria, the extended use of β-lactam antibiotics (mainly cephalosporins) in the treatment of infections caused by (especially) Gram-positive bacteria selectively supported the growth of β-lactamase-producing Gram-negative bacteria [9]. The problem of β-lactam antibiotic resistance became evident once ESBL-producing Gram-negative bacteria began to greatly decrease (or, to some extent, diminish) the effectiveness of broad-spectrum antibiotics, such as third-generation cephalosporins [9]. Additionally, ESBL-producing bacteria often display MDR traits, greatly complicating antimicrobial treatment against them [8,9]. These MDR traits have made ESBL-producing Gram-negative bacteria a top priority of international research efforts against antibiotic resistance and in drug development. Incidence of ESBLs and changes in their resistant trends especially due to their effect on the potency of widely used cephalosporins made them two interconnected topics. Because the increase in the prevalence of ESBLs can decrease the effectiveness of cephalosporins and support the spread of cephalosporin resistance, the usage of these antibiotics must be closely governed/monitored to minimise ESBL-selective isolations [1,10].

The first ESBL, an SHV variant (sulfhydryl reagent variable), was identified in Germany in 1983 [11]. Along with SHV, TEM (named after Temoniera, a Greek patient in whom the variant was isolated for the first time) and CTX-M are the most important and most frequently isolated variants of ESBLs [1,11,12,13,14]. To date, more than 200 subvariants of TEM and SHV have been identified [1,11].

Recent reports by the Centers for Disease Control and Prevention (CDC) on antibiotic resistance categorise the ESBL-producing *Escherichia coli* and *Klebsiella pneumoniae* as serious threats to healthcare systems worldwide [15,16,17]. In 2017 alone, nearly 9100 deaths of hospitalised patients were reported in the US as caused by these bacteria [15].

Despite reports from the CDC and the European Centre for Disease Prevention and Control (ECDC) on trends of resistance (conferred by ESBL), interpretations of the available data on the local scale are quite challenging and rather limited. This is because, firstly, the prevalence of ESBLs can differ according to the species, hospital allocation, geographical region, source of infection, and type of infection acquisition (nosocomial/community) [3,16,17], and secondly, the mentioned (CDC, ECDC) reports are very selective for the species of bacteria, and quite limited to the numbers of antibiotics and national centres monitored, making them insufficient for interpretating local complex drug-resistance profiles and phenotypic distributions.

Clinical studies on trends in the antibiotic resistance of ESBLs in Slovakia are missing. Such data provide important insights into the rationale for antibiotic stewardship policies for the country and region.

This study presents a comprehensive report from 3-year surveillance (2021 to 2023) of changes in prevalence of phenotypical variants, trends of antimicrobial resistance, and association of changes in resistant trends with the use of antibiotics, in ESBL-producing *E. coli*, *Proteus mirabilis*, and *K. pneumoniae* from the largest university hospital in Slovakia.

## 2. Results

### 2.1. Patient Characteristics and Demographic Data

A total of 74,900 patients were hospitalised during the study period at University Hospital Bratislava, Ružinov (23,977 patients in 2021; 25,472 patients in 2022; and 25,451 patients in 2023).

**Figure 1:**
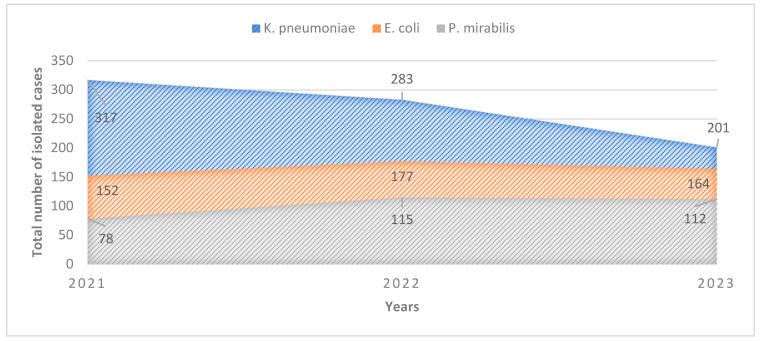
Graphical demonstration of the trends in isolation of ESBL-producing bacteria during the study period.

Data, including incidences of acute infections, phenotypic variant distributions, prevalence of phenotypical variants, and trends in antibiotic resistance by ESBL-producing *E. coli*, *P. mirabilis*, and *K. pneumoniae* isolates (hereafter ESBL-b), were collected because of their notably higher prevalence in comparison to other ESBL-producing bacteria isolates. A total of 1599 ESBL-b samples were isolated during the study period (547 isolated samples in 2021, 575 isolated samples in 2022, and 477 isolated samples in 2023). Female patients were infected more by ESBL-b than male patients in a nearly 2-to-1 ratio for every given year (Table 1). The incidence of ESBL-producing *E. coli* infections among hospitalised patients was 0.63% in 2021, 0.69% in 2022, and 0.64% in 2023. The incidence of ESBL-producing *K. pneumoniae* infections was 1.32% in 2021, 1.11% in 2022, and 0.79% in 2023. The incidence of ESBL-producing *P. mirabilis* infections was 0.33% in 2021, 0.45% in 2022, and 0.44% in 2023. We recorded a notable decline in the number of isolated ESBL-producing *K. pneumoniae* from 2021 to 2023 (Figure 1). The number of isolated ESBL-producing *E. coli* remained steady throughout the study period. We recorded a slight increase in the number of isolated ESBL-producing *P. mirabilis* (Figure 1). It is noteworthy that for all three ESBL-b groups, the number of isolated samples in 2023 was lower than the number of isolated samples in 2022, presenting a general decline in their occurrence.

The total recorded healthcare-associated infection (HAI) was 54% of all cases. Although we recorded an annual decrease in the number of HAI cases, the proportion of HAI did not decline (Table 1). The total associated-mortality-rate of ESBL-b infections (defined as the effect of infection on patient mortality, as an outcome, in association with other comorbidities) was 15% from 2021 to 2023. This means that 15% of patients diagnosed with ESBL-b infection died during the same hospitalisation. It is important to mention that the value of associated-mortality-rate is generally highly dependent on the type of infection, age of patient, and patient comorbidities. Bloodborne ESBL-b infections had a significantly higher percentage of associated-mortality-rate in comparison to other infections (Table 2).

### 2.2. Distribution of Samples’ Isolation

**Figure 2:**
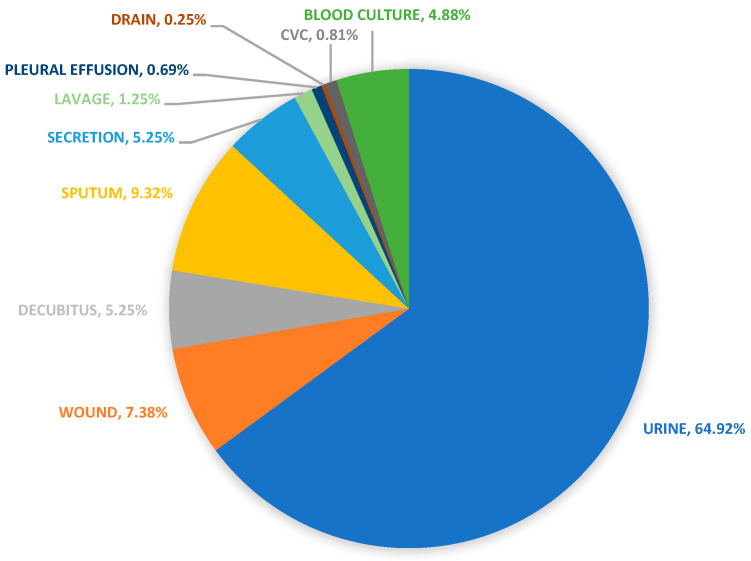
Graphical presentation of data on the sources of isolated samples during the study period.

The ESBL-b samples were predominantly isolated from urinary tract infections (UTIs) (64.92% of isolated cases), followed by chest and respiratory tract infections (16.51% of isolated cases), wound sample isolations (12.88% of isolated cases), and, finally, bloodborne infections (5.69% of isolated cases) (Table 3 and Figure 2).

Data on the distributions of isolated samples from ESBL-producing *E. coli*, *P. mirabilis*, and *K. pneumoniae* for each year are presented separately in Supplementary Figures S1–S9. Secretion refers to samples collected from upper respiratory tracts by active suction in patients under artificial pulmonary ventilation. Drain refers to samples collected from the drainage of wounds from hospitalised patients after surgery (i.e., surgical draining) or wounds requiring active drainage (i.e., wound draining).

We recorded increases in the trends in respiratory tract infections and decubitus isolations in ESBL-producing *P. mirabilis* during the study period. The percentage of collected respiratory tract infection isolates rose from 11.54% in 2021 to 34.82% in 2023 (Supplementary Figures S1–S3).

Predominantly, UTIs were the main complication caused by ESBL-producing *E. coli*. Despite the slight increase in the number of sample isolates from decubitus, we did not record notable changes in the distributions during the study period for *E. coli* as we registered for *P. mirabilis.* It is important to mention that bloodborne infections caused by ESBL-producing *E. coli* remained one of the most frequent complications in this bacterium (Supplementary Figures S7–S9). A high associated-mortality-rate was recorded among patients with bloodborne infections (Table 2). This signifies the importance of the changes in the trends of incidence of infections and antimicrobial resistance of ESBL-producing *E. coli,* which exert a significant associated-mortality-rate on diagnosed patients.

Although the highest associated-mortality-rate was recorded for bloodborne infections caused by ESBL-producing *E. coli* (n: 24/30, 80%), the associated-mortality-rate of these infections was high for the other two bacteria as well. In all, *K. pneumoniae* imposed the highest associated-mortality-rate (56% of the total collective associated-mortality-rates), with nearly the same associated-mortality-rates from bloodborne infections in comparison with *E. coli* and the highest associated-mortality-rates for respiratory tract infections (Table 2).

### 2.3. Trends in Antibiotic Resistance

Among the included ESBL isolates, we monitored the prevalences of the three main phenotypic variants, namely, SHV enzymes 2, 3, 4, 5, and 6; TEM enzymes 3, 4, 8, 9, 14, 15, 19, and 24; and CTX-M. We recorded the presence of aminoglycoside acetyltransferases (AGL AACs), phenotype (6′)-I, and AGL ANT (nucleotidyltransferase) phenotype (2′)-I, for the inclusion of cross-resistance to other antimicrobial families. Thus, for the cross-resistance, we only included data from variants with a significant number of isolates.

#### 2.3.1. Trends in Antibiotic Resistance of ESBL-Producing *E. coli*

**Figure 3:**
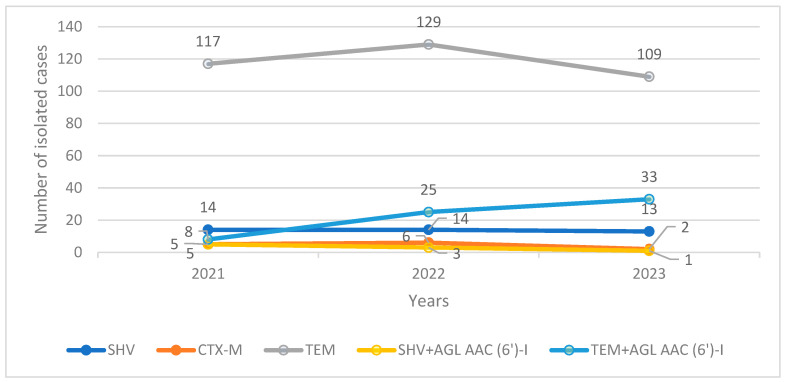
Trends in the numbers of isolated phenotypic variants of ESBL-producing *E. coli*.

The isolated variants of *E. coli* with the highest numbers were TEM and TEM + AGL AAC (6′)-I. We recorded the presence of cross-resistant phenotypes among TEM and SHV with AGL AAC (6′)-I (Figure 3). The three-year prevalences of SHV, CTX-M, and SHV + AGL AAC (6′)-I remained low throughout the study period (SHV: n/N: 41/493, 8.31%; SHV + AGL AAC (6′)-I: n/N: 9/493, 1.82%; CTX: n/N: 13/493, 2.63%). Only the TEM + AGL AAC (6′)-I variant increased in prevalence between 2021 (n/N: 8/496, 1.61%) and 2023 (n/N: 33, 6.65%) (Figure 3). The numbers of the other variants of *E. coli* were statistically insignificant and were not included in the study’s data set.

A susceptibility test was performed on a panel of twenty antibiotics from seven antibiotic families in a routine antibiogram examination of the bacterium.

**Figure 4:**
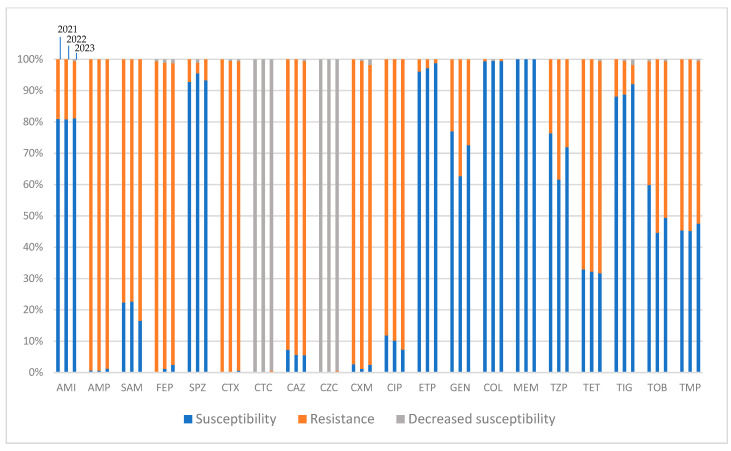
Three-year changes in the antibiotic resistances of isolated ESBL-producing strains of *E. coli* (all phenotypes). AMI: amikacin; AMP: ampicillin; CAZ: ceftazidime; CIP: ciprofloxacin; COL: colistin; CTX: cefotaxime; CTC: cefotaxime–sulbactam; CXM: cefuroxime; CZC: ceftazidime–sulbactam; ETP: ertapenem; FEP: cefepime; GEN: gentamicin; MEM: meropenem; SAM: ampicillin–sulbactam; SPZ: sulperazone (cefoperazone–sulbactam); TET: tetracycline; TIG: tigecycline; TMP: trimethoprim–sulfamethoxazole; TOB: tobramycin; TZP: Tazocin (piperacillin–tazobactam).

Each column in Figure 4 shows the percentages of the susceptibility vs. resistance (or decrease in sensitivity) of the bacterium to a given antibiotic over a year. For example, the three columns above amikacin represent the changes in *E. coli*’s resistance to this antibiotic in 2021 (left column), 2022 (middle column), and 2023 (right column). Decreased susceptibility means the need for a higher dose/exposure time (than normally recommended) of a given antibiotic for it to be effective against a given bacterium.

The families of antibiotics included the following: penicillins (ampicillin, ampicillin–sulbactam, and piperacillin–tazobactam), cephalosporines (cefotaxime, cefotaxime–sulbactam, ceftazidime, ceftazidime–sulbactam, cefuroxime, cefepime, and cefoperazone–sulbactam), carbapenems (ertapenem and meropenem), glycyclines (tigecycline), fluroquinolones (ciprofloxacin), aminoglycosides (amikacin, gentamicin, and tobramycin), tetracyclines (tetracycline), sulphonamides (trimethoprim–sulfamethoxazole), and polymyxins (colistin).

We recorded high resistance rates (RRs) for ESBL-producing *E. coli* to penicillins (RR ampicillin: 99%; RR ampicillin–sulbactam: 84%) (Figure 4). Among cephalosporins, cefoperazone–sulbactam sustained a remarkable 7% RR without significant changes in the trends of resistance. As for the rest of the cephalosporins, the RRs remained as high as 96% on average. Cefotaxime–sulbactam and ceftazidime–sulbactam sustained steady decreases in their sensitivity statuses, meaning the need for greater exposure (time/dose) of them in order to exert their effectivity (Figure 4). Colistin and carbapenems neared a 0% RR among the isolated strains. Rises in RRs were recorded for gentamicin (*p* = 0.525) and tobramycin (*p* = 0.077) in 2023 in contrast with 2021. Although the RRs decreased slightly in 2023 (in comparison to 2022 for aminoglycosides), the new RRs were still higher than those in 2021, indicating a trend of increasing resistance to these antibiotics by the bacterium. In spite of all this, none of the changes in the three-year trends in the RRs of the bacterium were statistically significant.

For a better demonstration of the effect of the different ESBL phenotypes on cephalosporin resistance, an individual resistance trend analysis was performed for every phenotypic variant (Supplementary Figures S10–S13). Moreover, the resistance traits in the presence of the AGL AAC (6′)-I phenotype for changes in aminoglycoside susceptibility and cross-resistance to fluroquinolones were separately analysed for the TEM variants (Supplementary Figure S10 demonstrates the resistance trends for the TEM variant isolates without the presence of the AGL AAC (6′)-I phenotype, and Supplementary Figure S11 demonstrates the resistance trends for the TEM variant isolates with the presence of the AGL AAC (6′)-I phenotype concomitantly).

We recorded significant changes in the trends in resistance of the TEM + AGL AAC (6′)-I variants to tobramycin during the study period (*p* < 0.001). Moreover, these variants were 100% resistant to ciprofloxacin (in comparison with the TEM variants) as a cross-resistant trait in the presence of the AGL AAC (6′)-I phenotype. Despite the increase in the RR to gentamicin in these strains, the changes were not statistically significant (*p* = 0.84). Of note, the TEM variants showed a generally higher RR to gentamicin (average RR of 35.16% vs. average RR of 4.36%) in comparison with the strains with the AGL AAC phenotype. The opposite, however, was seen for the RR to amikacin between the two variants, where the average RR of the TEMs was 4.52% and the average RR of the TEM + AGL AAC was 85.54% (Supplementary Figures S10 and S11).

#### 2.3.2. Trends in Antibiotic Resistance of ESBL-Producing *P. mirabilis*

**Figure 5:**
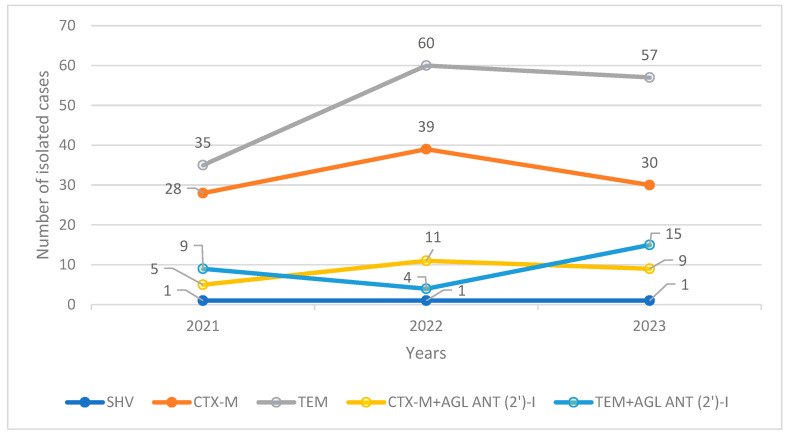
Trends in the numbers of isolated phenotypic variants of ESBL-producing *P. mirabilis*.

The highest numbers of isolated variants of *P. mirabilis* were found for TEM and CTX-M. We recorded the presence of cross-resistant phenotypes among the TEM and CTX with AGL ANT (2′)-I in this bacterium (Figure 5). The three-year prevalences of the TEM/CTX-M variants with AGL ANT (2′)-I phenotype remained near 10% (8.20% for CTX + AGL ANT (2′)-I and 9.18% for TEM + AGL ANT (2′)-I). Except for TEM + AGL ANT (2′)-I, the other isolated variants showed a mild declining trend in the number of isolations for 2023 in comparison with 2022. However, the total numbers of isolated cases for all variants (except SHV) were higher than those in 2021 (Figure 5).

**Figure 6:**
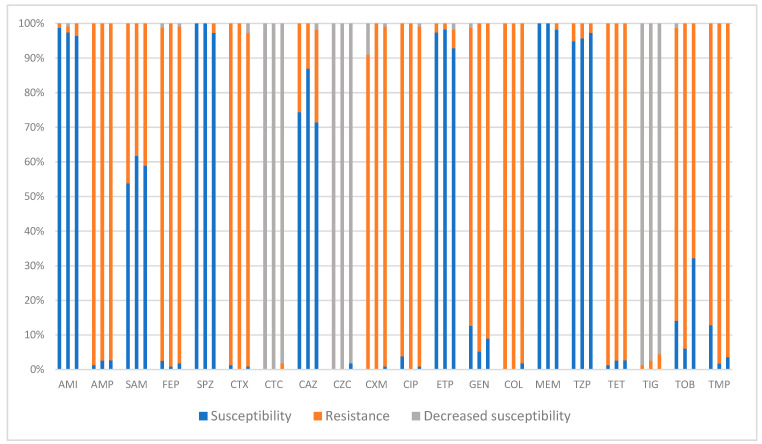
Three-year changes in the antibiotic resistances of isolated ESBL-producing strains of *P. mirabilis* (all phenotypes). AMI: amikacin; AMP: ampicillin; CAZ: ceftazidime; CIP: ciprofloxacin; COL: colistin; CTX: cefotaxime; CTC: cefotaxime–sulbactam; CXM: cefuroxime; CZC: ceftazidime–sulbactam; ETP: ertapenem; FEP: cefepime; GEN: gentamicin; MEM: meropenem; SAM: ampicillin–sulbactam; SPZ: sulperazone (cefoperazone–sulbactam); TET: tetracycline; TIG: tigecycline; TMP: trimethoprim–sulfamethoxazole; TOB: tobramycin; TZP: Tazocin (piperacillin–tazobactam). Complementary information for better interpretation of the figure is provided in the text following Figure 4.

Cefoperazone–sulbactam sustained a notable <1% RR (0.89%) during the study period. Ceftazidime showed a lower RR in isolated *P. mirabilis* strains compared with *E. coli* (21.82% vs. 93.67%) (Figure 4, Figure 5 and Figure 6). The average three-year resistance rate to colistin was 99.40%. The bacterium showed much higher resistances to gentamicin and tobramycin compared with *E. coli* (average RR of 90.64% vs. 29.25% for gentamicin and average RR of 82.13% vs. 48.28% for tobramycin). On the contrary, the RR toward amikacin was markedly lower in *P. mirabilis* isolates (average RR of 2.2% vs. 18.86% in *E. coli*). Interestingly, we documented a statistically significant drop in RR of the bacterium to tobramycin in 2023 in comparison to 2021 or 2022 (*p* = 0.008). Other than this, none of the other changes in the three-year trends in the RRs of the bacterium were statistically significant. The resistance traits for TEM phenotype are presented in Supplementary Figure S14. The resistance traits in the presence of the AGL ANT (2′)-I phenotype for changes in aminoglycoside susceptibility and cross-resistance to fluroquinolones were analysed in the TEM variants (presented in Supplementary Figure S15). As expected, higher RRs were recorded for the TEM + AGL ANT (2′)-I variants to aminoglycosides, except for amikacin. Despite the minor increase in the RR to amikacin in 2023 for the TEM + AGL ANT (2′)-I variants, the average three-year RRs stayed markedly low (Supplementary Figure S15).

#### 2.3.3. Trends in the Antibiotic Resistance of ESBL-Producing *K. pneumoniae*

**Figure 7:**
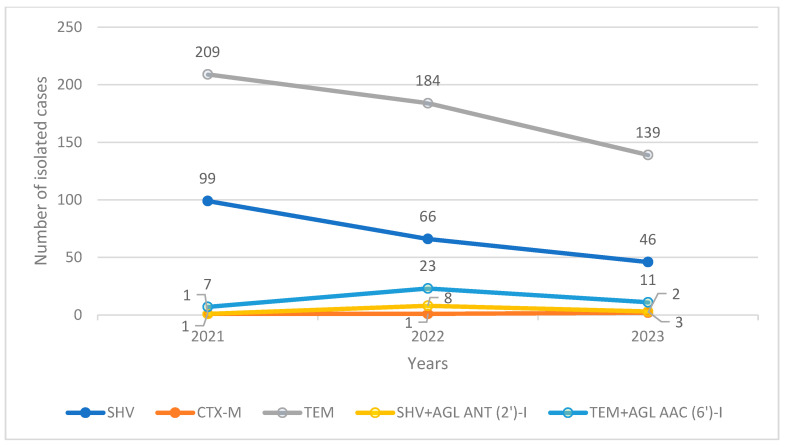
Trends in the numbers of isolated phenotypic variants of ESBL-producing *K. pneumoniae*.

Isolated phenotypic variants of *K. pneumoniae* showed a declining trend over the study period. Cross-resistance was recorded for the SHV and TEM variants in the presence of the AGL AAC (6′) and ANT (2′)-I phenotypes. The numbers of combined isolated and cross-resistant samples, however, remained too low for any meaningful statistical analysis (Figure 7). The isolated variants that were the highest in number were TEM (the same as *E. coli* and *P. mirabilis*), followed by the SHV variants. This demonstrates the clear differences in phenotypic distribution among the three bacteria. Although the number of isolated samples of the TEM variants exhibited a declining trend, their prevalence remained nearly the same in 2021 and 2023 (n/N: 209/317, 66% vs. n/N: 139/201, 69%). Meaning even if the total number of ESBL-producing *K. pneumoniae* isolates was lower in a given year, nearly 70% of all isolates in that year were still of the TEM variant. The prevalence of the SHV variants, however, mildly declined from 31% (n/N: 99/317) in 2021 to 23% (n/N: 46/201) in 2023, most likely due to the increase in the distributions of the TEM and TEM + AGL AAC (6′)-I variants (Figure 7).

**Figure 8:**
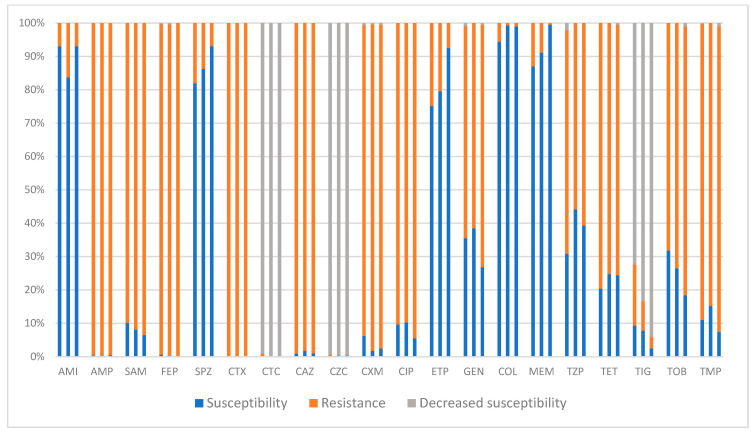
Three-year changes in the antibiotic resistances of isolated ESBL-producing strains of *K. pneumoniae* (all phenotypes). AMI: amikacin; AMP: ampicillin; CAZ: ceftazidime; CIP: ciprofloxacin; COL: colistin; CTX: cefotaxime; CTC: cefotaxime–sulbactam; CXM: cefuroxime; CZC: ceftazidime–sulbactam; ETP: ertapenem; FEP: cefepime; GEN: gentamicin; MEM: meropenem; SAM: ampicillin–sulbactam; SPZ: sulperazone (cefoperazone–sulbactam); TET: tetracycline; TIG: tigecycline; TMP: trimethoprim–sulfamethoxazole; TOB: tobramycin; TZP: Tazocin (piperacillin–tazobactam). Complementary information for better interpretation of the figure is provided in the text following Figure 4.

Although multidrug-resistant statuses were found for ESBL-producing *E. coli* and *P. mirabilis* isolates as well, the worst RR to antibiotics still belongs to *K. pneumoniae* (Figure 8). On the basis of the data presented, many of the isolated samples from ESBL-producing *K. pneumoniae* were categorised as XDR bacteria. More concerning is the isolation of strains with resistance to colistin and/or meropenem, which are the last-standing effective antibiotics against the bacterium (Figure 8). The only good news is that the RR trends did not increase for either of the mentioned antibiotics. On the contrary, we documented a statistically significant drop in RRs of the bacterium to ertapenem (*p* < 0.001) and meropenem (*p* < 0.001) in comparison between years 2021 and 2023. Individual cephalosporin-resistance trend analyses were performed on the TEM and SHV variants (Supplementary Figures S16 and S17). An analysis of the cephalosporin-resistance trends or cross-resistances to aminoglycosides–fluroquinolones were not performed because of the low numbers of isolated cases and statistical insignificance of the other variants.

As for penicillins and cephalosporins, the recorded RRs of the bacterium remained at over 90% for all of the tested antibiotics, except for piperacillin–tazobactam (with an RR of 60%), sulperazone, and cefotaxime/ceftazidime–sulbactam combinations (Figure 8). The resistance rate of sulperazone decreased from almost 20% in 2021 to less than 10% in 2023, making the antibiotic one of the few potent treatments available against ESBL-producing *K. pneumoniae*. Cefotaxime–sulbactam and ceftazidime–sulbactam (similar to *E. coli*) showed a constant decrease in sensitivity with no recorded resistance.

### 2.4. Changes in Drug Consumption Trends

**Figure 9:**
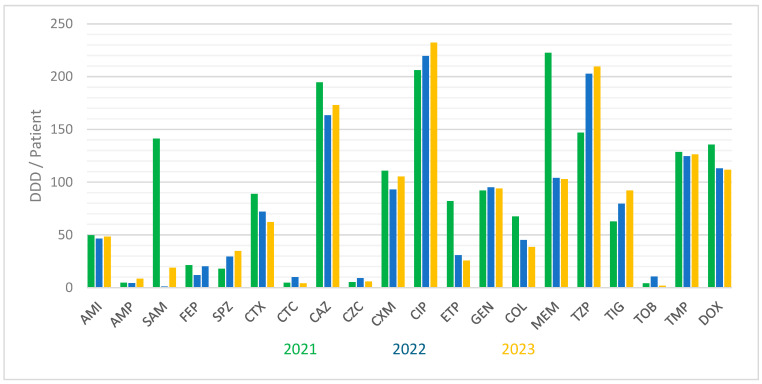
Three-year changes in estimated drug consumption based on defined daily dose/patient. AMI: amikacin; AMP: ampicillin; CAZ: ceftazidime; CIP: ciprofloxacin; COL: colistin; cefotaxime; CTC: cefotaxime–sulbactam; CTX: cefotaxime; CXM: cefuroxime; CZC: ceftazidime–sulbactam; DOX: doxycycline; ETP: ertapenem; FEP: cefepime; GEN: gentamicin; MEM: meropenem; SAM: ampicillin–sulbactam; SPZ: sulperazone (cefoperazone–sulbactam); TIG: tigecycline; TMP: trimethoprim–sulfamethoxazole; TOB: tobramycin; TZP: Tazocin (piperacillin–tazobactam).

The drug consumption rate was determined using an anatomical therapeutic chemical classification (ATC) system with the implementation of the defined daily dose (DDD). To enable statistical comparison and in accordance with local hospital regulations, we used DDD/patient indicators for the determination of estimated antibiotic consumption based on World Health Organization (WHO) recommendations [18] (Figure 9).

The highest consumption rates were recorded for ciprofloxacin, ceftazidime, and piperacillin–tazobactam. The greatest decreases in medication consumption (between 2021 and 2023) were seen for ampicillin–sulbactam, ertapenem, and meropenem, followed by colistin, cefotaxime, and ceftazidime (Figure 9). A simple linear regression test (considering the limitation of available data) was used to calculate the significance of changes in trends of antibiotic consumption over the years. We recorded a statistically significant drop in the consumption rate for sulperazone (R^2^ = 0.988, slope: −60.96, *p* = 0.002 and CL: 95%), meropenem (R^2^ = 0.998, slope: −60.33, *p* = 0.012 and CL: 95%), and ertapenem (R^2^ = 0.996, slope: −18.67, *p* = 0.029 and CL: 95%), between 20221 and 2023. No other recorded declining consumption rates were statistically significant (ceftazidime: R^2^ = 0.167, slope: 3.33, *p* = 0.875 and CL: 95%, cefotaxime: R^2^ = 0.988, slope: −7.33, *p* = 0.097 and CL: 95%). Increases in consumption rates were recorded for piperacillin–tazobactam (R^2^ = 0.833, slope: 16.50, *p* = 0.417 and CL: 95%), tigecycline (R^2^ = 0.900, slope: 10, *p* = 0.333 and CL: 95%), and ciprofloxacin (R^2^ = 0.867, slope: 8, *p* = 0.400 and CL: 95%). None of the recorded increasing trends of consumption were statistically significant.

The data on consumption trends (based on DDD/patient) of tetracycline are presented as doxycycline consumption in Figure 9, as it is the sole tetracycline currently available in Slovakia.

## 3. Discussion

### 3.1. Patient Demography, Incidence, Health-Associated Infection Rate, and Overall Associated-Mortality-Rate

Demographically, female patients were infected nearly twice as much with ESBL-b in our study (n/N: 1010/1599, 63% vs. n/N: 589/1599, 37%; Table 1). This can partially be explained by the possibly higher occurrence of UTIs among female patients (as the overall highest infection type recorded) and the higher percentage of females in the country with a slightly older age category compared to male patients. Other than the number of isolated infections, there were no other notable differences among the two cohorts of patients.

The changes in the incidence trends in the ESBL-producing *E. coli* and *P. mirabilis* infections exhibited steady mild growth over the study period (0.63% in 2021 vs. 0.64% for ESBL-producing *E. coli*; 0.33% in 2021 vs. 0.44% in 2023 for ESBL-producing *P. mirabilis*; Figure 1). The incidence of ESBL-producing *K. pneumoniae* infections exhibited a declining trend, dropping from 1.32% in 2021 to 0.79% in 2023, nearly reaching the incidence recorded for ESBL-producing *E. coli* and ESBL-producing *P. mirabilis* infections for the same year (Table 1). The high incidence of ESBL-producing *K. pneumoniae* infections in 2021 corresponds to post-COVID-19 bacterial epidemy (seen worldwide) resulting from the overuse of antibiotics and long hospitalisations during the pandemic [19,20,21,22].

Our data show a worrying 54% HAI among the ESBL-b isolates. Despite the decrease in the number of total ESBL-b samples, the rate of HAIs remained above 50% in 2023. It is only thanks to the low incidence of ESBL-b infections that the number of diagnosed HAIs was below 300 patients per 20,000 hospitalisations a year. Because of the limited ability to filter out colonisations and/or imported infections in published studies, we opted to abandon the comparison of the percentage rates to avoid introducing data set bias. Nonetheless, the importance of the recorded high HAI in relation to associated mortality or morbidities should not be undermined, especially as improvements in the staff’s/facility’s hygienic/epidemiologic standards may result in a decrease of as much as half of recorded infections without the need for antibiotics.

The associated-mortality-rate is an epidemiological index for the estimation of disease burden. This index, however, is highly dependent on patients’ comorbidities and infection types [23]. For example, the associated-mortality-rate recorded for UTIs in our study is almost 5.49% (n/N: 57/1038, Table 2), whereas the associated-mortality-rate recorded for bloodborne infections (BBIs) is 74.72% (n/N: 68/91, Table 2). The high associated-mortality-rates for ESBL-b in BBIs and respiratory tract infections (RTIs) have been documented in many studies [24,25,26]. This large difference in associated-mortality-rates in relation to infection type is especially important considering the shift in the distribution of isolated samples. This means that if the same bacteria in future years cause more BBIs or RTIs rather than UTIs, we can expect a surge in associated-mortality-rates and healthcare burden. Unfortunately, such a shift is already recorded in our data set. Respiratory tract infections, notably, became the second most common infections in 2023, replacing wound infections in 2021 and 2022 in ESBL-producing *P. mirabilis* (Supplementary Figures S1–S6). The difference between the associated-mortality-rates recorded for wound infections and RTIs (Table 2) in this bacterium is large enough to consider this shift in infection trend to be a serious epidemiologic alert.

A rise in the prevalence of respiratory tract infections in European countries post-COVID-19 has been recorded [27,28]. Both the WHO and ECDC reported increases in the prevalence of RTIs due to high co-circulating viral and bacterial pathogens in populations after the COVID-19 pandemic, with expected increasing trends in the future [3,28,29]. Other than the mentioned worldwide epidemiologic shift in the distribution of infection type, phenotypic variations in the ESBLs in our study did not show any significant associations with such changes. Genetic strain typing (as a limitation) was not performed in our study. A continuation of the rise in the prevalence of these infections can be expected due to easy patient-to-patient transfer (via respiratory droplets), the increase in associated complications (length of hospitalisation and/or treatment, healthcare-associated financial burden, and patient death), and the lack of emplacement of proper policy and epidemiological management.

### 3.2. Associated-Mortality-Rate, Prevalence of Phenotypic Isolates, Trends in Phenotypic Distribution, and Antibiotic Resistance in ESBL-Producing E. coli

The highest associated-mortality-rate in our cohort of patients was recorded for *E. coli* BBIs (80%, Table 2). The European Centre for Disease Prevention and Control, in their 2023 report on antimicrobial resistance surveillance in Europe, categorise *E. coli* as a “major cause” of bloodstream infections on the continent [16]. The ECDC’s four-year study (2016–2020) on antimicrobial resistance burden in European countries reported that the largest problem arises from BBIs caused by *E. coli* strains resistant to third-generation cephalosporins, in terms of both the number of cases and associated mortality [30,31].

Although most of the recent published data did not find a direct connection between BBIs caused by ESBL-producing *E. coli* and short-term mortality (30 days), they were in agreement on the effect of these infections on the significant increase in the length of hospitalisation and/or the rate of infection re-occurrence [23,26,31,32].

In our study, TEM and TEM + AGL AAC (6′)-I (hereafter TEM+) were the most frequently isolated variants of ESBL-producing *E. coli* (Figure 3). Only the TEM+ variant among all of the phenotypes showed a trend of increasing isolations, reaching more than four times the number of isolations in 2023 compared to 2021 (Figure 3). Although the TEM phenotype is commonly found to be a major ESBL variant of *E. coli* worldwide, the distribution of other variants is quite variable for different countries and continents. Many studies from East Asian, Southeastern European, and African countries in recent years have documented the increasing trend in the isolation of the CTX-M variant as the most common or second most common phenotype after TEM [11,14,15,33,34]. In our study, however, such a trend was not recorded. In fact, the numbers of other phenotypic isolations (namely, CTX-M, SHV, and SHV + AGL AAC (6′)-I) were too low to be statistically significant in terms of the prevalence or effect on antibiotic resistance. In spite of this, because the epidemiological distribution of phenotypic variants can differ greatly [7], sometimes even among health facilities in the same city, the data presented on such surveillances must be interpreted cautiously in regard to the generalisation of the phenotypic presentations. These epidemiological data are important not only because they present the current local phenotypic distribution of the different variants, but they can provide information on the main changes in the distribution trends and the predicted associated antibiotic resistances in the future. In this sense, the significances of the phenotypic presentations and changes in their occurrence are clinically translated into changes in antibiotic resistance and antibiotic susceptibility.

The antibiotic resistance profile of the bacterium demonstrates the presence of MDR strains. This is because, except for carbapenems, colistin, sulperazone, tigecycline, and (partially) aminoglycosides, the bacterium showed high resistance to the “majority” of the 12 other antibiotics tested. Moreover, except for meropenem, no other antibiotic (including colistin and ertapenem) showed 0% resistance to the bacterium throughout the study period. This means that at least one XDR strain (resistant to ertapenem and/or colistin) was isolated in both 2021 and 2023.

Among cephalosporins, only sulperazone with an RR of less than 10% and, “partially”, ceftazidime–sulbactam and/or cefotaxime–sulbactam with decreased susceptibilities, demonstrated efficacy against the bacterium. This good susceptibility rate, however, was notably lower in the SHV phenotype isolates, which demonstrated a higher percentage of resistance to sulperazone (up to 30% as the only effective cephalosporin) and complete resistance (or decreased susceptibility) to all other tested cephalosporins (Supplementary data, Figure S12). These data suggest that any changes in the isolation trends in the SHV phenotype in the future may significantly increase the resistance of the bacterium against sulperazone and general cephalosporins.

Among the aminoglycosides, amikacin showed a good susceptibility rate (more than 80%, Figure 4) to isolated *E. coli* in our study. The only exception was documented for the TEM+ *E. coli* variants, for which almost 100% resistance to the antibiotic was recorded in 2021 and 2022 (100% and 98% resistant rates, respectively). These high resistance rates, however, abruptly dropped in 2023, decreasing the resistance rates of these bacteria to amikacin to nearly 60%. Such a decrease in resistance to amikacin was concomitant with a significant increase in resistance to tobramycin over the same year (increasing from a resistance rate of less than 10% in 2022 to more than 90% in 2023, *p* < 0.001, Supplementary Figure S11). Such an abrupt change in the trends in aminoglycoside resistance in the ESBL phenotypic category could be due to populational changes among genetical strains. The reasons for such an assumption are, firstly, because of the increase in the prevalence of TEM+ phenotype in 2022 and 2023, suggesting the introduction of a new strain among the tested population, and, secondly, due to the lack of recorded changes in amikacin or tobramycin consumption dynamics in those years (Figure 9).

Moreover, all of the TEM+ isolates showed 100% resistance to ciprofloxacin due to known cross-resistance abilities in the presence of the AGL AAC (6′)-I phenotype [35], making the variant the most antibiotic-resistant phenotype among the ESBL-producing *E. coli* strains.

To put it all into context, *E. coli,* as the second most frequently isolated ESBL-b in our study, showed a mild but (concerningly) constant increase in isolation rate trends, a high associated mortality/morbidity burden, and consistent MDR and occasional XDR traits. Although no overall significant changes in the trends in antibiotic resistance were recorded, treatment of the bacterium remains challenging because, except for good susceptibility to last-resort antibiotics (meropenem–ertapenem, piperacillin–tazobactam), highly cytotoxic antibiotics (colistin, gentamicin, and amikacin), and antibiotics with poor serum concentrations (i.e., not suitable for endovascular treatments such as tigecycline), the recorded susceptibility to other “first-choice” antibiotics was disturbingly low. In light of the increase in the isolation rates of TEM+ variants with higher resistance traits to aminoglycosides and fluroquinolones, this might become even worse over the coming years. For the time being, sulperazone with a (documented) [1,8,36,37] high susceptibility rate can be considered a viable “first” option in the treatment of life-threatening infections caused by MDR/XDR ESBL *E. coli*. Importantly, however, one should always consider that the success rate of sulperazone can be variable depending on the disease severity and comorbidities of patients [1]. Furthermore, the antibiotic’s rates of consumption vs. resistance of the antibiotic should be cautiously monitored because of its effectivity as a last-resort treatment for some “hard to treat” carbapenem-resistant bacteria (such as the XDR *A. baumannii* [38]) and the omittance of the antibiotic-supported isolation of the SHV variants.

### 3.3. Associated-Mortality-Rate, Prevalence of Phenotypic Isolates, Trends in Phenotypic Distribution, and Antibiotic Resistance of ESBL-Producing P. mirabilis

The only bacterium in our study with notable increases in the annual number of isolations and incidence was *P. mirabilis* infections. The increase in the incidence of this bacterium in recent years has been documented worldwide [39,40,41]. This change in incidence of infection corresponds to the shift recorded in infection isolation type of the bacterium, mentioned earlier.

The change in the distribution of the isolation types from wound infections to RTIs (especially in 2023) was recorded for *P. mirabilis* (Supplementary Figures S1–S3). As discussed before, the shift in the isolation-type trends could be the result of several epidemiological, phenotypic, and genotypic determinants. Whatever the cause, however, the importance of such changes is demonstrated in the healthcare-associated burdens (HABs) that the bacteria impose in the form of associated-mortality-rates. Although *P. mirabilis* is not a common cause of complicated, life-threatening infections in the general population, it has been connected to health-associated urinary tract, respiratory tract, and bloodstream infections in immunosuppressed patients [39,40,41]. Despite the lowest recorded associated-mortality-rate, the shift in isolation-type trends, along with the increase in incidence of infection, may greatly affect the HABs that the bacterium imposes in the coming years. The ageing population in Slovakia and increase in the number of geriatric patients, along with the increase in the rate of RTIs (especially due to the previously mentioned high associated-mortality-rates), signify the importance of monitoring of changes in the RRs and associated-mortality-rates of this bacterium in the country. Globally, however, still UTIs followed by wound infections are the most common complications caused by this bacterium.

Phenotypically, the TEM variant, followed by the CTX-M variant, was the most common isolated phenotype at our hospital (Figure 5). The numbers of the other isolated variants, namely, TEM + AGL ANT (2′)-I, CTX-M + AGL ANT (2′)-I, and SHV, remained steadily low and statistically insignificant. Only the TEM + AGL ANT (2′)-I phenotype showed a mild and negligible increase in the number of isolations during the study period.

Looking at the trends in the antibiotic resistances of the bacteria, a clear contrast of the patterns of resistance among *P. mirabilis*, *E. coli*, and *K. pneumoniae* is shown. Firstly, natural known resistances to colistin, tigecycline, and tetracycline [42,43] were recorded (as expected) for isolated *P. mirabilis* (Figure 6). Secondly, the highest resistances to gentamicin and tobramycin were documented for this bacterium in our study, rendering these antibiotics completely useless in our hospital. High resistances to gentamicin and tobramycin have been reported in recent years, especially in clinical settings [2,44]. Worryingly enough, these resistance trends have significantly increased worldwide over the last two years [44]. Despite these reports and the presence of high RR of the bacterium to these antibiotics in our data set, we recorded a significant decrease in RR to tobramycin in 2023. This may suggest a possible shift in the trends of resistance of the bacterium to this antibiotic in the coming years.

Carbapenems, piperacillin–tazobactam, sulperazone, and amikacin were the only five antibiotics with recorded RRs less than 10%, followed by ceftazidime with almost 30% and ampicillin–sulbactam with close to 50%. All of the other tested antibiotics showed significantly low RRs to the bacterium, demonstrating MDR traits by ESBL *P. mirabilis* (Figure 6). The most concerning, however, was the lack of a single antibiotic in the year 2023 that had 100% susceptibility to the bacterium’s isolated strains (Figure 6). This means that we isolated strains of ESBL *P. mirabilis* that were resistant even to the mentioned five “last-resort” antibiotics with low RRs. These data confirm the presence of XDR strains of ESBL *P. mirabilis* circulating among the bacterium’s population. The high antibiotic-resistance profile, along with the increase in the incidence of infections and virulence patterns of the bacterium in relation to shifts in the isolation type, demonstrates the concerning changes in the epidemiological dynamics of the bacterium today and in the coming years. The absence of significant changes in the trends of resistance, in addition to the high rates of susceptibility to the selected antibiotics and low associated-mortality-rates, so far, are the only positive conclusions from the presented data.

### 3.4. Associated-Mortality-Rate, Prevalence of Phenotypic Isolates, Trends in Phenotypic Distribution, and Antibiotic Resistance in ESBL-Producing K. pneumoniae

The documented associate-mortality-rate of BBIs caused by ESBL-producing *K. pneumonia* is as high as ESBL-producing *E. coli* [26]. Unlike ESBL-producing *E. coli, however,* a direct association between the bacterium’s BBIs and short-term mortality risk has been documented [17]. Higher virulence is seen not only in BBIs caused by ESBL-producing *K. pneumoniae,* but also in other types of infection caused by the bacterium (in comparison to ESBL-producing *E. coli* and/or *P. mirabilis*) [17,26,45]. This may explain the overall higher associated-mortality-rate recorded for ESBL-producing *K. pneumoniae* (56%, Table 2) in our study.

Moreover, the MDR/XDR phenotypes are more common among *K. pneumoniae* strains, making its treatment more complicated [17]. Although the increase in the prevalence of carbapenem-resistant (CR) strains due their XDR traits and ability to transfer resistance genes is currently the most concerning topic related to the bacterium [46], the importance of ESBL-producing strains due to their higher isolation rates and healthcare-associated burdens remains clear. Based on the 2023 ECDC report on antibiotics surveillance in Europe, *K. pneumoniae* strains resistant to third-generation cephalosporins (along with *E. coli* and MRSA) had the largest burden of disease over a four-year period from 2016 to 2020, “generating 58.2% of the total burden as measured in disability-adjusted life years” [16]. All this, in light of the bacterium’s highest incidence of infection (in our cohort of patients), denotes the great clinical significance of *K. pneumoniae* in the epidemiological mapping of ESBL-b’s healthcare-associated burdens and in antimicrobial policy in Slovakia.

TEM and SHV were the most frequently isolated variants in ESBL-producing *K. pneumoniae* in our study (Figure 7). Although most of the recently published data suggest an increase in the prevalence of the CTX-M phenotype worldwide [8,47,48], the regional variability of the phenotypic dominance, especially from Northern African, Middle Eastern, and Eastern European countries, showed higher TEM isolations [12,49,50]. This suggests that the prominence of TEM over CTX-M varies by geographical region and/or study population. Interestingly, the CTX-M variant in our hospital was isolated so rarely (same as *E. coli*) that the number of isolations could not provide any statistically significant information on antimicrobial-resistance trends (Figure 7). As mentioned earlier, the concomitant presence of the AGL phenotypes ANT (2′)-I with the SHV variants and of AAC (6′)-I with the TEM variants was recorded. However, similar to the CTX-M phenotype, the number of isolations was insufficient for inclusion in the statistical analysis (Figure 7). Neither of the variants increased in prevalence during the study period, ensuring a declining trend in the bacterium’s isolation pattern.

Analysing the trends in the antibiotic resistance of the bacterium provides valuable clinical information. Most importantly, we did not see any statistically significant increases in the RRs to any of the tested antibiotics (mainly antibiotics with effectivity against the bacterium). This shows that the bacterium’s resistance did not markedly improve during monitoring period. On the contrary, we recorded a statistically significant decrease in the resistance rate of isolated *K. pneumoniae* to carbapenems between 2021 and 2023 (from approximately 35% to less than 10% for ertapenem, *p* < 0.001, and from approximately 12% to 0% for meropenem *p* < 0.001, from 2021 to 2023). It is noteworthy that we recorded a statistically significant decline in the usage of meropenem (R^2^ = 0.998, slope: −60.33, *p* = 0.012 and CL: 95%) and ertapenem (R^2^ = 0.996, slope: −18.67, *p* = 0.029 and CL: 95%) during the same period. In fact, this was our only recorded correlation between the changes in drug consumption and RRs among the three bacteria. This could suggest that the changes in epidemiological situations after the pandemic and decrease in the number of lengthy hospitalisations, along with changes in antibiotic policy and significant decline in the use of carbapenems, contributed to this change in the RRs.

As previously mentioned, the presented data do not undermine the power of the antibiotic resistance of the bacterium. First, colistin-resistant strains were isolated every year throughout the study period. This is especially important in the context of CR strains, where colistin is considered to be one of the effective last-resort antibiotics available. Secondly, a 0% resistance rate to any of the antibiotics was not observed in *K. pneumoniae* (Figure 8). This highlights that every year, despite the predominance of MDR strains, there was at least one XDR strain isolated. The MDR/XDR strains of the ESBL-producing *K. pneumoniae* have been reported globally. Different studies from the USA, Asia, and Europe document the emergence of XDR strains in this bacterium for both the CTX-M and TEM variants [49,50]. These studies illustrate the critical issue surrounding the treatment of infections caused by the bacterium and its burden on healthcare systems worldwide.

Moreover, except for amikacin with an RR of less than 10%, the bacterium showed high resistance to the other tested aminoglycosides. The implementation of amikacin as an alternative antibiotic in the treatment of ESBL-producing *K. pneumoniae,* especially in the case of carbapenem’s usage restrictions (due to resistance or unavailability), has been suggested. For example, in a study evaluating the efficacy of amikacin (vs. its toxicity) in the treatment of UTIs caused by ESBL-producing MDR/XDR *E. coli* and/or *K. pneumoniae*, the authors reported that the antibiotic had an overall bactericidal success rate of 97.1%, with negligible nephrotoxicity [51]. Hence, they concluded that amikacin can be considered before carbapenems as an alternative treatment in the mentioned infection settings once the bacterium is resistant to the other oral antibiotics [51]. It is not necessary to mention that the results of this study (and similar publications) should be evaluated cautiously in regard to the study design and patient cohorts. For example, in the mentioned study, the majority of patients had no or negligible nephrological complications, whereas the application of the antibiotic in the setting of poly-morbid, immunosuppressed hospitalised patients with chronic kidney disease could lead to different results.

Among cephalosporines, only sulperazone with a decrease in RR during the study period (from almost 20% in 2021 to less than 10% in 2023) remained a potent antibiotic in treatment against ESBL-producing *K. pneumoniae*.

In summary, the presented data show that *K. pneumoniae* was the most frequently isolated ESBL-producing bacteria, and despite the declining incidence of infection during the study period, the bacterium remained the major cause of ESBL-b-induced infections in our hospital. The importance of these data is especially evident considering the bacterium’s high RRs. Only 5 (last-resort antibiotics) out of the 20 antibiotics tested faced a 10% resistance from the bacterium (Figure 8), leaving the remaining 15 antibiotics facing (at least) more than 55% resistance rates. Moreover, among those five effective antibiotics, one strain of the bacterium showed resistance to all of them in every given year. This constant presence of MDR and/or XDR strains, along with highest recorded incidence of infection and associated-mortality-rate, raises the alarm for the urgent implementation of strict monitoring programmes and antimicrobial stewardship in the country against ESBL-producing *K. pneumoniae*. Especially when changes in antibiotic consumption (specifically carbapenems) show clear associations with the decline in the resistance trends of the bacterium, the management of the allowed defined daily doses can greatly contribute to influencing projected resistance trends in the near future. In this sense, the usage of susceptible antibiotics should be restricted in accordance with the minimum inhibitory concentration (MIC) of a given isolated bacterium in relation to the severity of the infection and current local antibiotic-resistance trends.

For the time being, the usage of sulperazone or amikacin as possible first-choice antibiotics and carbapenems and colistin as possible second-choice antibiotics in the treatment of severe life-threatening infections caused by MDR or XDR ESBL *K. pneumoniae*, under the previously mentioned considerations, seems to be our last viable option.

### 3.5. Is It Time to Start Worrying? New Strategies and Treatment Options

Our results, in accordance with the global picture of ESBL-b, demonstrate the presence of MDR-XDR strains of bacteria isolated at our hospital, with susceptibility to only one-quarter of the antibiotics tested on them. We demonstrated that strains resistant to those few antibiotics already existed and were circulating in our clinical setting. Concerns about these resistance strains are even more tangible when observing the shift in the isolation trends of the bacteria and expected increases in their associated-mortality-rates in the coming years. Hence, it is, indeed, time to start worrying.

The growing concerns about the increase in the antibiotic resistance and decrease in available treatment options for MDR bacteria (including ESBL-b) are not a secret. Since 2001, the WHO has emphasised in different publications the growing problem of antimicrobial resistance worldwide [52]. In 2017, a so-called “priority pathogen list” was published by the WHO, listing the most dangerous bacteria for which new antibiotics are urgently needed [53]. Both ESBL-producing *E. coli* and *K. pneumoniae* were among the critical priority bacteria listed [53]. Unfortunately, however, not much has changed since the publication of the list. Despite national and international efforts in the management of the problem with the development of intercontinental antibiotic stewardship initiatives and international investments in different sectors related to antimicrobial resistance programmes, we have neither new antibiotics nor declining trends in the prevalence of the bacteria worldwide.

It is a known fact that since the last decades of the previous century, we have not been able to develop new molecules effective against Gram-negative bacteria [54]. What we have developed so far are only combinations of known antibiotics with newer versions of beta-lactamase inhibitors. Molecules such as ceftazidime–avibactam, ceftolozane–tazobactam, meropenem–vaborbactam, and imipenem–cilastatin–relebactam are some of these new, complex last-resort antibiotics. Although these antibiotics are typically reserved for severe or complicated infections, where resistance to other treatments is a concern, several reports on the development of resistance by ESBL-b against them have already been documented. For example, the Infectious Disease Society of America (IDSA), in their recent guidelines, published in 2021, on the treatment of ESBL-b, CR Enterobacterales, and difficult-to-treat *P. aeruginosa*, reports the emergence of resistances by ESBL *E. coli* and *K. pneumoniae* to ceftazidime–avibactam and ceftolozane–tazobactam, especially under the selective pressure of other antibiotics [55]. In another study, Ibaideya et al. reported the development of resistance to meropenem–vaborbactam by ESBL-b through mutations at the target site of the antibiotic and the overproduction of efflux pumps [44]. These publications and the results of several similar studies demonstrate the growing resistance against these last-resort antibiotics, implying the insufficient power of the antibiotic arsenal available and emphasising the need for cautious antimicrobial usage, ongoing monitoring, and development of new therapeutic strategies.

Improvement of hygienic/epidemiologic standards to decrease the HAI and new treatment modalities, such as phage therapy, antimicrobial peptides (AMPs), CRISPR-Cas systems, immunotherapies, and especially the development of monoclonal antibodies and probiotic/microbiome manipulations, are some of these strategies.

Phage therapy as a treatment modality in the management of MDR bacteria has gained attention for many years. Specifically, the target selectiveness and ability to penetrate biofilm are great advantages of this methodology. However, like antibiotics, the emergence of bacteria resistant to phages has been reported [56]. Moreover, the strict specificity of the phages to the bacterial strain targeted narrows their potency to a limited number of bacteria, creating the need for the implementation of a phage cocktail to broaden their efficacy [56,57].

Unlike bacteriophages, AMPs can target a wide range of pathogens, including bacteria, fungi, and viruses, and low bacterial resistance has been reported for them due to their mechanism of action [58,59]. Additionally, some AMPs can modulate the immune system, enhancing the body’s natural defences against infections, and synergically, they can be used with traditional antibiotics [59]. However, the instability of AMPs under physiological conditions and proteolytic degradation by host enzymes decrease their therapeutic potential [59]. For this reason, designing a stable AMP with the possibility of its effective delivery to the site of infection to minimise the degradative effects of body enzymes is quite challenging [59].

The CRISPR-Cas system has been explored as a potential novel treatment modality in the management of MDR bacteria in recent years [60]. The possibility of engineering a system to target and destroy specific bacterial DNA would make them highly selective [60,61]. This additionally provides the theoretical potential for the design of a bacteria-resistant proof system, which can be adapted to target various bacterial strains by altering the guide RNA [61]. However, similar to AMPs, the delivery of CRISPR-Cas components into bacterial cells within the human body is still a significant hurdle [61]. Phages are being investigated as potential carriers; however, the efficacy of this method varies greatly among different bacterial strains [61]. In addition, there is always the risk of off-target effects, whereby unintended DNA sequences might be cut, potentially leading to unintended consequences [61].

Immunotherapies, particularly the development of monoclonal antibodies (mAbs), have shown to be promising in the treatment of drug-resistant bacteria [62]. Tailor-made antibodies specific to a given bacterial strain and the enhancement of the immune system and its potential combination with antibiotics and other immune-modulating agents make them an attractive alternative in the management of MDR and XDR infections [62]. Despite its cost and unavailability, the method is still cheaper than many of the therapeutic options listed above and more reproducible. However, like other methods, immunotherapy has its disadvantages as well. Because mAbs are antibodies, the body can produce antigens against them, making them ineffective [62]. Moreover, they are strain-limited and functional for the specific strains of bacteria for which they are made [62], yet the modality holds very promising potential in the treatment of complicated drug-resistant infections once other methods are no longer effective. Additionally, it has theoretical application capacity in the treatment (decolonisation) of MDR-XDR carriers. However, more studies and research are needed to evaluate the method’s efficacy and safety [62].

All in all, despite the development of many potential therapeutic methodologies, today, the only known effective treatment remains antibiotics. To further support their effectiveness for the foreseeing future, usage management, strict antimicrobial stewardship programmes developed on the basis of local data, and continuous monitoring of resistance trends are crucial for this challenging battle against drug-resistant bacteria.

## 4. Materials and Methods

### 4.1. Healthcare Facilities, Patients, and Specimens

In this observational, retrospective study, we monitored and gathered data from all clinically relevant bacterial isolates sampled from patients presenting signs and/or symptoms of an infectious disease for whom antibiotic sensitivities were tested. Data were gathered from sample isolates of patients hospitalised in any of the hospital’s inpatient wards or departments, including all surgical departments, the internal disease department, anaesthesiology department, and all corresponding intensive care units of University Hospital Bratislava, Ružinov, for a period of three years (from the beginning of 2021 to the end of 2023). We excluded data from outpatient wards/departments, repetitive sample isolates, and/or bacterial colonisation without the presence of an active infectious disease with elevated inflammatory markers. The majority of hospitalised patients were from the Bratislava region, representing about 10% of Slovakia’s population. For each isolate, we collected information on the sampling location and antibiogram.

### 4.2. Isolation and Proof of Bacterial Strains

Sterile swabs were used to collect samples from the nose, tonsils, wounds, and decubitus. While swabbing may present limitations in wound infection identification, each case included in our study (as mentioned above) involved patients with clinical signs consistent with infection (including erythema, swelling, and discharge at the wound site). Clinical assessment by the attending physicians documented in patients’ files, coupled with elevated inflammatory markers supported the classification of all included cases as active infections rather than colonisation. Moreover, local protocols were followed for sample analysis (included bacterial load thresholds and qualitative assessment, especially for samples from sputum) to infer infection rather than colonisation. Blood sampling was performed using a peripheral intravenous puncture. Urine was collected by midstream urine sampling or via a urinary catheter. All samples of body fluids (ascites and pleural effusion), sputum, lavage, upper respiratory tract secretions (presented as secretion in the text), operational wound drainage, and pus were collected using standardised aseptic techniques. The samples were processed using conventional methods approved by the Slovak Ministry of Health. Strain identification was performed after classical isolation and classical biochemical and cultivation methods using a Bruker MALDI Biotyper (Bruker, Billerica, MA, USA), as well as the application of the MALDI-TOF Mass Spectrometry method where applicable [63]. Minimum inhibitory concentration (MIC) tests, using the disc diffusion technique, were performed on isolated bacteria. The antibiotic testing results, as well as the ESBL identification, were interpreted according to EUCAST guidelines (European Committee on Antimicrobial Susceptibility Testing) [64]. A panel of antibiotics for testing was predetermined by diagnostic laboratories, defined for Gram-negative and -positive isolates. Further, diagnosed isolates underwent phenotypic evaluation using colorimetric susceptibility tests and a bacterial suspension inoculum (Mueller–Hinton broth) with a MidiTech20 analyser [65]. Incidence of infection, phenotypic characteristics, prevalence of phenotypical variants, and antimicrobial resistance changes were monitored for ESBL-producing *E. coli*, *P. mirabilis* and *K. pneumoniae* because of their higher incidences and statistical significances compared to other isolated bacteria in this category. We included data from three main phenotypic ESBL variants, namely, TEM 3, 4, 8, 9, 14, 15, 19, and 24; SHV 2, 3, 4, 5, and 6; and CTX-M. Data on the concomitant presence of the AGL AAC (6′)-I and ANT (2′)-I phenotypes with the main ESBL variants were included to study cross-resistance to aminoglycosides and fluroquinolones. Clinical relevance was defined as samples isolated from patients with clinical and laboratory presentations of active infectious processes in whom previous colonisation with the isolated bacterium had not been documented. Multidrug resistance was defined as the nonsusceptibility of the bacterium to ≥1 agent in ≥3 antimicrobial categories [66]. Extensive drug resistance was defined as nonsusceptibility to ≥1 agent in all but ≥2 antimicrobial categories [66]. Healthcare-associated infection was defined as the infection that patient acquires while receiving medical care in a healthcare facility occurring 48 h after his/her admission [67]. Associated-mortality-rate was defined as associated burden of infectious disease as the cause/or aiding cause of death during the same hospitalisation. The data of both HAI and associated-mortality-rate were gathered from medical files of the included patients in the study during their hospitalisation. Defined daily dose was designated as the assumed average of the maintenance dose per day for a drug. The anatomical therapeutic chemical classification system was used for drug coding based on the WHO database [18]. After determination of the ATC/DDD, the internal hospital consumption data were used to calculate the consumption of antibiotics in DDD per hospitalised patient (DDD/patient indicator) for comparative statistical purposes [18].

### 4.3. Statistical Analysis

The data collected during the monitoring period were analysed using Microsoft Excel (version 16.88). Statistical significance of changes in RRs of a bacterium to an antibiotic between different years was assessed using chi-squared (level of significance of *p* value was set as *p* ≤ 0.05, with degree of freedom (*df*) of 1). A simple linear regression test was used to calculate the significance of changes in trends of antibiotics’ consumption over surveilled years. The *p*-value was determined using a *t* distribution with n − 2 degrees of freedom (*df*), and the confidence level of the *p*-value was determined to be 95%. Prevalence of phenotypical variants (in text used as prevalence) was defined as the number of a given isolated phenotypical variant of an ESB-producing bacteria, divided by the total number of the same ESBL-producing isolated bacteria for a defined period of time. Incidence was defined as the number of (given) isolated ESBL-producing bacteria (isolated from patients with clinical presentation of an active infectious process), divided by the total number of hospitalised patients for a defined period of time.

### 4.4. Ethical Consent

The study and its methodology were carefully reviewed by the Ethics Committee, which undertook a comprehensive assessment in line with the Health Care Act No. 576/2004 Col., Section 26, Paragraph 1. The committee evaluated the research’s adherence to ethical standards and legislative requirements, with particular focus on the retrospective and observational nature of the study, the use of anonymized and aggregated data, and the lack of direct patient interaction or health interventions. Given these factors, the study did not require individual consent or explicit ethical approval under Slovak legislation. This statement regarding the ethical consent status of the study has been documented in the Institutional Board Review.

## 5. Conclusions

Despite the steady incidences of infection caused by the tested bacteria, the changes in the trends in infection isolation types and high HAI are concerning. Based on the shifts in isolation type, increases in the associated-mortality-rates of *K. pneumoniae* and *P. mirabilis* are expected in the coming years. This is especially worrying considering the presence of MDR strains among all three groups, in addition to the proven existence of XDR strains among the circulating bacteria at our hospital. Except for meropenem on ESBL-producing *E. coli,* no other antibiotic showed 100% susceptibility to the tested bacteria over the study period. This high drug-resistance profile emphasises the urgent need for clinical-based antimicrobial stewardship programmes and continued monitoring of resistance trends in the country. New achievable treatment strategies, such as immunotherapy along with controlled antibiotic usage and improvement in staff/environmental hygienic policies in light of the existence of resistance to last-resort antimicrobials, should be encouraged. Due to insufficiencies in nation-wide databases, further comprehensive multicentre studies in Slovakia are needed to better map the phenotypic distribution and trends in antibiotic resistance of the bacteria in the country.

## Figures and Tables

**Table 1:** Number of isolated ESBL bacteria and their incidence of infection, associated-mortality-rate, and percentage of healthcare-associated infections.

	Total	2021	2022	2023
Number of hospitalised patients	74,900	23,977	25,472	25,451
Number of cases of isolated ESBL-producing *E. coli* (Incidence, %)	493	152 0.63%	177 0.69%	164 0.64%
Number of cases of isolated ESBL-producing *K. pneumoniae* (Incidence, %)	801	317 1.32%	283 1.11%	201 0.79%
Number of cases of isolated ESBL-producing *P. mirabilis* (Incidence, %)	305	78 0.33%	115 0.45%	112 0.44%
Total number of ESBL-b	1599	547	575	477
Number of male patients with isolated ESBL-b (n/N, cases%)	589/1599, 37%			
Age of male patients (Mean)	62.8 (SD ± 14.2)			
Number of female patients with isolated ESBL-b (n/N, cases%)	1010/1599, 63%			
Age of female patients (Mean)	64.3 (SD ± 17.8)			
Healthcare-associated infections of isolated ESBL-b (n/N) (%)	863/1599 54%	295/547 54%	291/575 50%	277/477 58%
Associated-mortality-rate of isolated ESBL-b (n/N) (%)	240/1599 15%	89/547 16%	91/575 16%	60/477 13%

ESBL: extended-spectrum β-lactamase; ESBL-b: ESBL-producing bacteria (including all three groups of bacteria: *E. coli*, *K. pneumoniae*, and *P. mirabilis*); N: total number of isolated samples; n: number of isolated samples for a given variable (for example, healthcare-associated infections (n/N) means the number of isolated healthcare-associated infection samples to the total number of isolated samples, presented as percentages); SD: standard deviation.

**Table 2:** Associated-mortality-rates of different ESBL-producing bacteria categorised on the basis of the type of infection.

	Total	UTI	RTI	W	BBI
AsMR of ESBL-producing *E. coli* (n/N) (%)	68/240 28%	30/378 8%	14/42 33%	0/43 0%	24/30 80%
AsMR of ESBL-producing *K. pneumoniae* (n/N) (%)	134/240 56%	14/490 2.8%	84/179 47%	1/88 1.1%	35/44 79%
AsMR ESBL-producing *P. mirabilis* (n/N) (%)	38/240 16%	13/170 7.6%	16/43 37%	0/75 0%	9/17 53%

AsMR: associated-mortality-rate; BBI: bloodborne infection; ESBL: extended-spectrum β-lactamase; N: total number of AsMRs for all three groups of bacteria over the three-year study period; n: number of AsMRs for a given bacteria over the three-year study period; RTI: respiratory tract infection; UTI: urinary tract infection; W: wound infection.

**Table 3:** Total distribution of the isolates sampled during the study period categorised on the basis of the type of infection and type of sample collected.

Type of Infection	Type of Sample: n/N, Percentage
Urinary tract infections	Urine: n: 1038/1599, 64.92%
Chest and respiratory tract infections	Sputum: n: 149/1599, 9.32%, Secretion: n: 84/1599, 5.25% Lavage: n: 20/1599, 1.25% Pleural effusion: n: 11/1599, 0.69%
Decubitus, wound, and operational wound drainages	Wounds: n: 118/1599, 7.38% Decubitus: n: 84/1599, 5.25% Drain: n: 4/1599, 0.25%
Bloodborne infections	Blood culture: 78/1599, 4.88% Central venous catheter (CVC): n: 13/1599, 0.81%

n: number of given infection isolations; N: total number of ESBL-b isolations.

## Data Availability

Data is contained within the article.

## Supplementary Materials

The following supporting information can be downloaded at: https://www.mdpi.com/article/10.3390/ph17111517/s1, Figure S1: Distribution of isolated samples of *P. mirabilis* 2023; Figure S2: Distribution of isolated samples of *P. mirabilis* 2022; Figure S3: Distribution of isolated samples of *P. mirabilis* 2021; Figure S4: Distribution of isolated samples of *K. pneumoniae* 2023; Figure S5: Distribution of isolated samples of *K. pneumoniae* 2022; Figure S6: Distribution of isolated samples of *K. pneumoniae* 2021; Figure S7: Distribution of isolated samples of *E. coli* 2023; Figure S8: Distribution of isolated samples of *E. coli* 2022; Figure S9: Distribution of isolated samples of *E. coli* 2021; Figure S10: Three-year changes in the antibiotic resistance to cephalosporins, aminoglycosides and fluroquinolones of isolated ESBL producing TEM variants in *E. coli*.; Figure S11: Three-year changes in the antibiotic resistance to cephalosporins, aminoglycosides and fluroquinolones of isolated ESBL producing TEM variants with presence of concomitant AGL AAC (6′)-I phenotype in *E. coli*.; Figure S12: Three-year changes in the antibiotic resistance to cephalosporins of isolated ESBL producing SHV variants in *E. coli*.; Figure S13: Three-year changes in the antibiotic resistance to cephalosporins of isolated ESBL producing CTX-M variants in *E. coli*.; Figure S14: Three-year changes in the antibiotic resistance to cephalosporins, aminoglycosides and fluroquinolones of isolated ESBL producing TEM variants in *P. mirabilis*.; Figure S15: Three-year changes in the antibiotic resistance to cephalosporins, aminoglycosides and fluroquinolones of isolated ESBL producing TEM variants with presence of concomitant AGL ANT (2′)-I phenotype in *P. mirabilis*.; Figure S16: Three-year changes in the antibiotic resistance to cephalosporins of isolated ESBL producing TEM variants in *K. pneumoniae*.; Figure S17: Three-year changes in the antibiotic resistance to cephalosporins of isolated ESBL producing SHV variants in *K. pneumoniae*.
