# Supplementary material for: Is It Time to Start Worrying? A Comprehensive Report on the Three-Year Prevalence of ESBL-Producing Bacteria and Their Trends in Antibiotic Resistance from the Largest University Hospital in Slovakia
Source: Pharmaceuticals (Basel). 2024 Nov 11;17(11):1517. doi: 10.3390/ph17111517 (PMC11597623; doi:10.3390/ph17111517)
Supplement: Supplementary file 1 [file pharmaceuticals-17-01517-s001.zip › pharmaceuticals-3272344-supplementary.pdf]

pharmaceuticals

MDPI

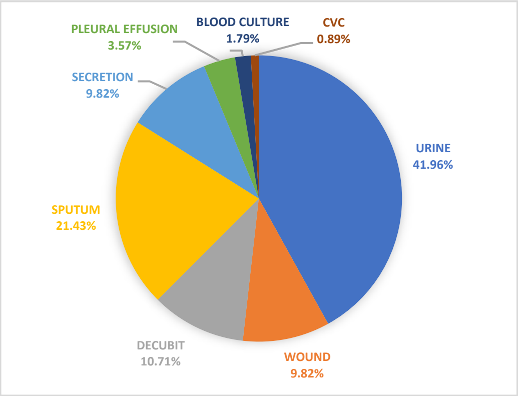

Figure S1. Distribution of isolated samples of *P. mirabilis* 2023.

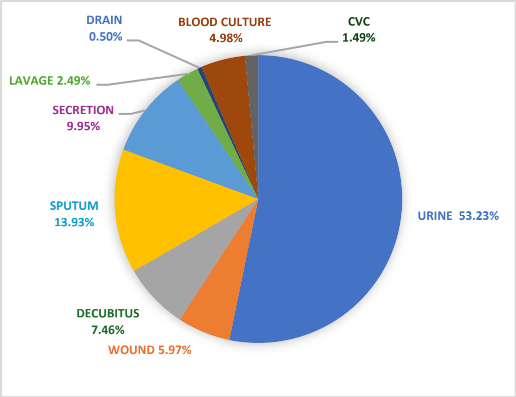

Figure S4. Distribution of isolated samples of *K. pneumoniae* 2023.

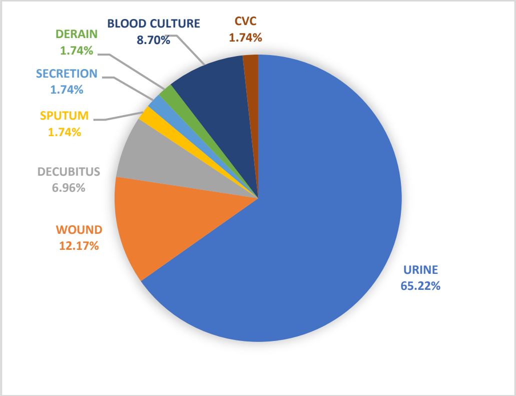

Figure S2. Distribution of isolated samples of *P. mirabilis* 2022.

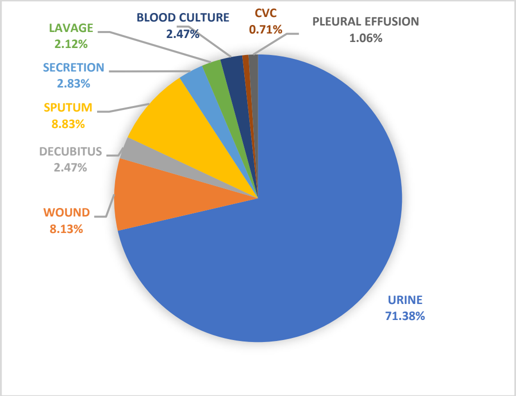

Figure S5. Distribution of isolated samples of *K. pneumoniae* 2022.

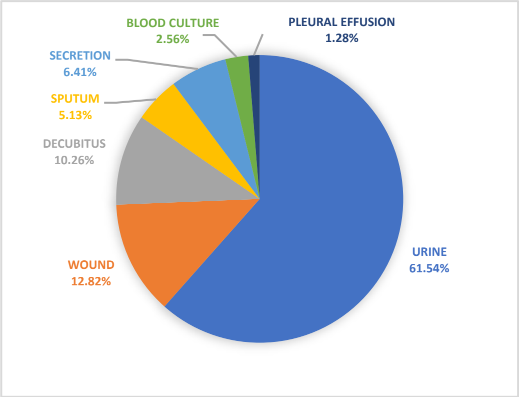

Figure S3. Distribution of isolated samples of *P. mirabilis* 2021.

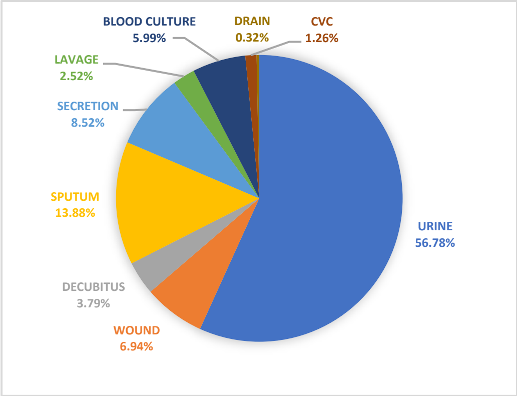

Figure S6. Distribution of isolated samples of *K. pneumoniae* 2021.

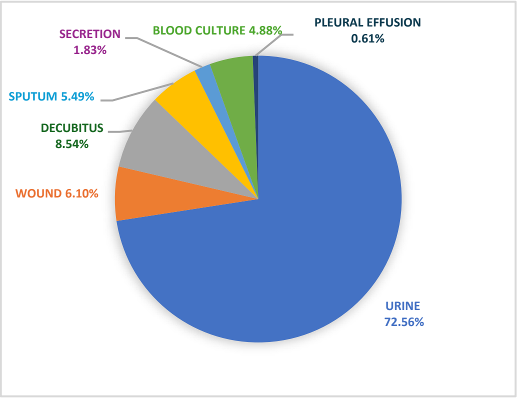

Figure S7. Distribution of isolated samples of *E. coli* 2023.

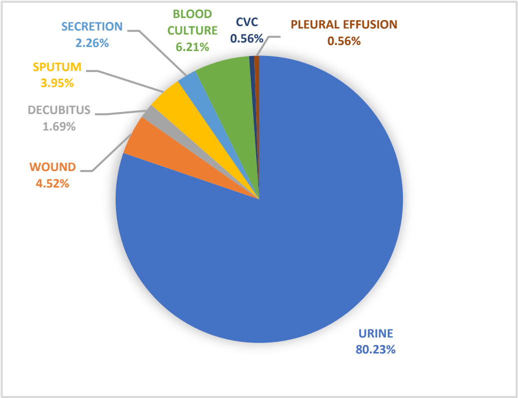

Figure S8. Distribution of isolated samples of *E. coli* 2022.

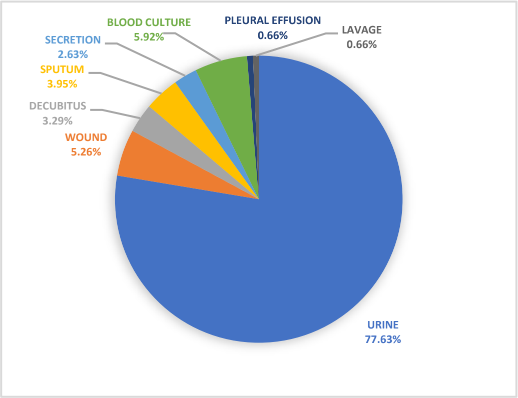

Figure S9. Distribution of isolated samples of *E. coli* 2021.

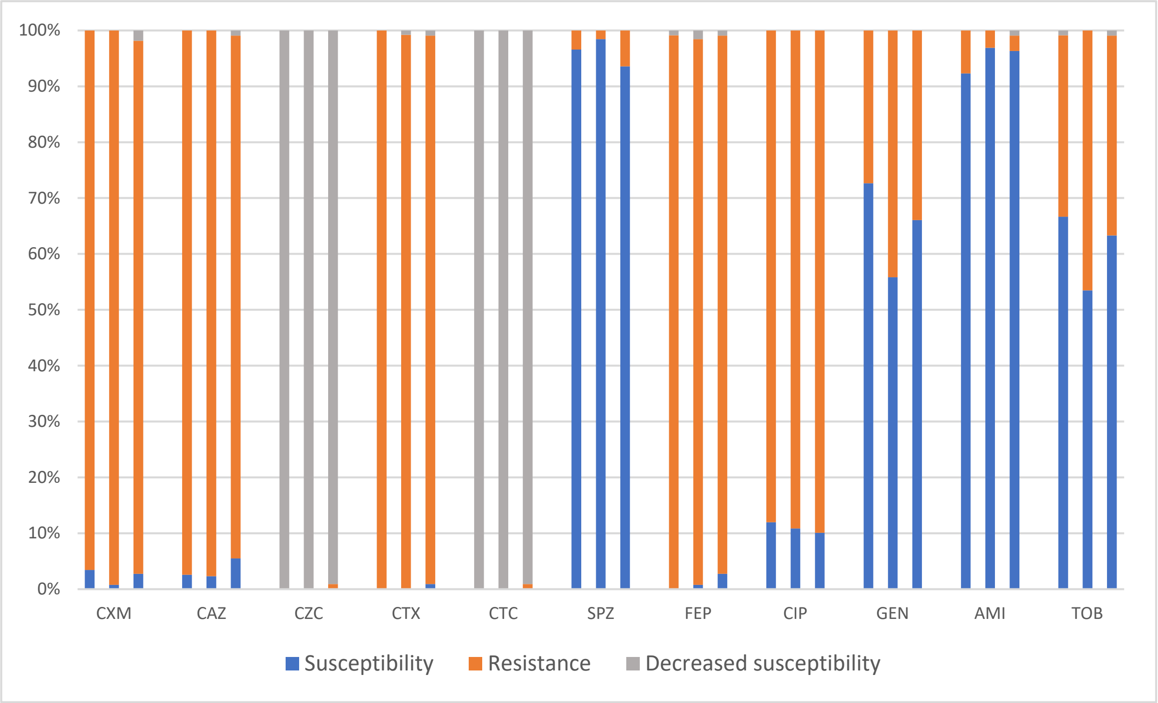

Figure S10. Three-year changes in the antibiotic resistance to cephalosporins, aminoglycosides and fluroquinolones of isolated ESBL producing TEM variants in *E. coli*. AMI: amikacin, CAZ: ceftazidime, CIP: ciprofloxacin, CTX: cefotaxime, CTC: cefotaxime-sulbactam, CXM: cefuroxime, CZC: ceftazidime-sulbactam, FEP: cefepime, GEN: gentamycin, SPZ: sulperazone (cefoperazone-

sulbactam), TOB: tobramycin. Complementary information for better interpretation of the figure above is provided in the text following Figure 4.

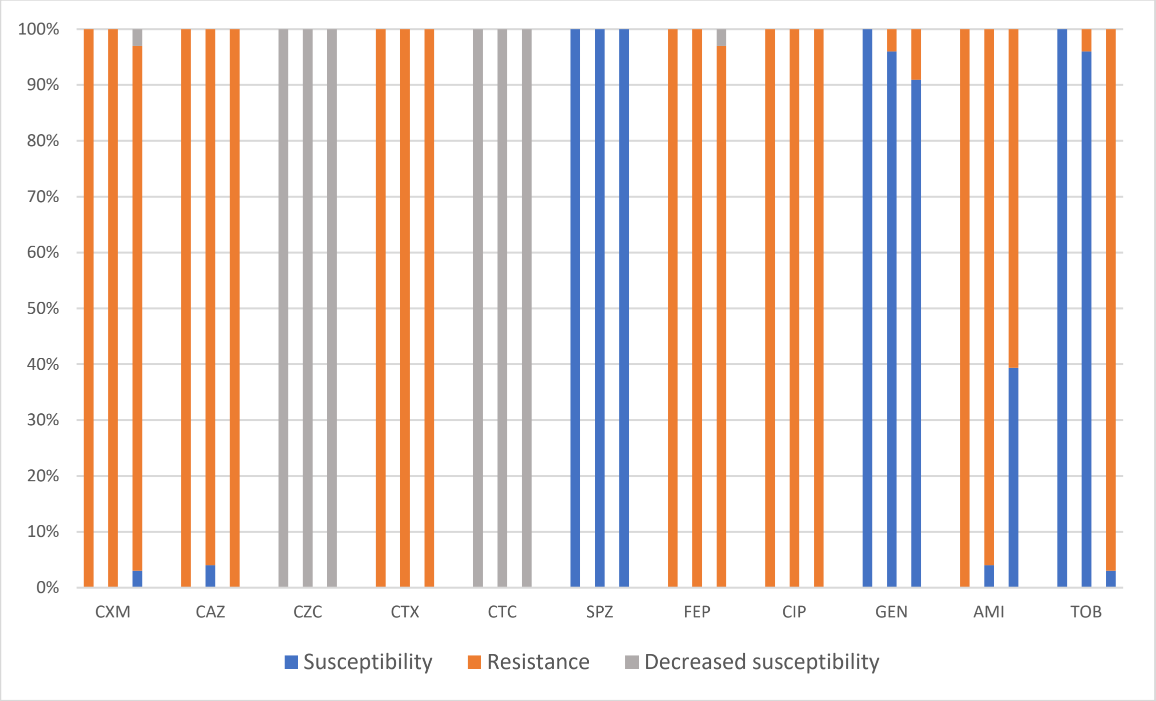

Figure S11. Three-year changes in the antibiotic resistance to cephalosporins, aminoglycosides and fluroquinolones of isolated ESBL producing TEM variants with presence of concomitant AGL AAC (6′)-I phenotype in *E. coli*. AMI: amikacin, CAZ: ceftazidime, CIP: ciprofloxacin, CTX: cefotaxime, CTC: cefotaxime-sulbactam, CXM: cefuroxime, CZC: ceftazidime-sulbactam, FEP: cefepime, GEN: gentamycin, SPZ: sulperazone (cefoperazone-sulbactam), TOB: tobramycin. Complementary information for better interpretation of the figure above is provided in the text following Figure 4.

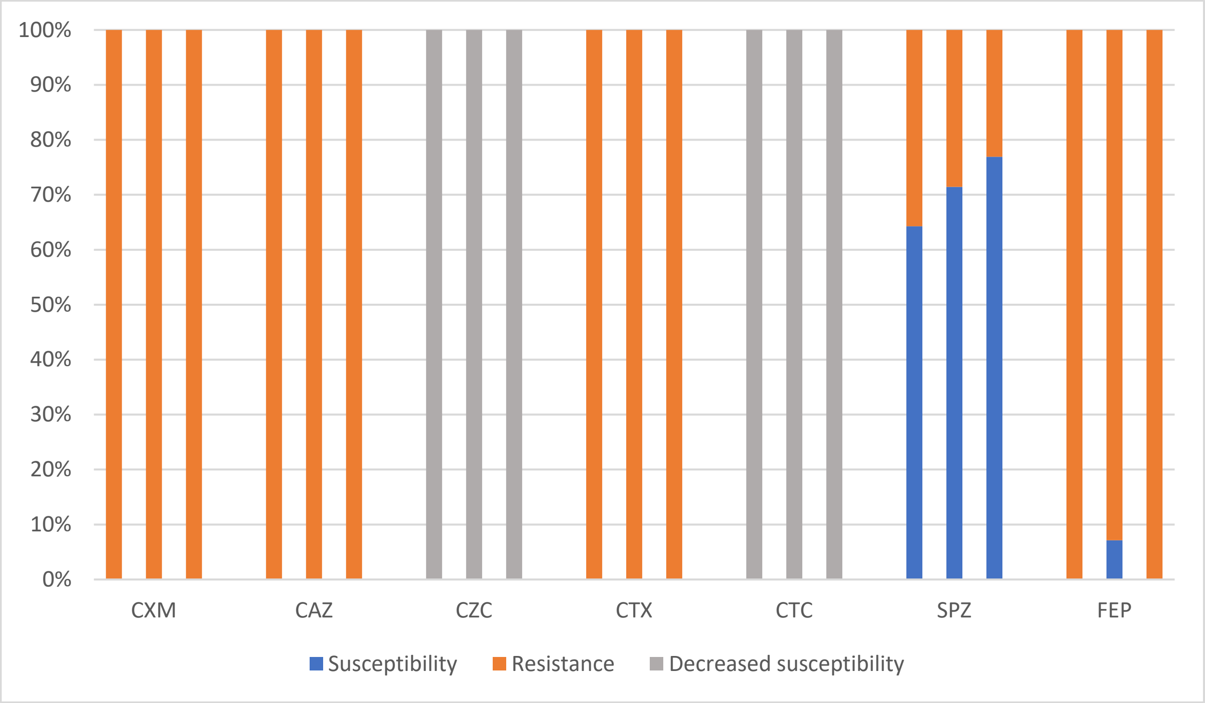

Figure S12. Three-year changes in the antibiotic resistance to cephalosporins of isolated ESBL producing SHV variants in *E. coli*. CAZ: ceftazidime, CTX: cefotaxime, CTC: cefotaxime-sulbactam, CXM: cefuroxime, CZC: ceftazidime-sulbactam, FEP: cefepime, SPZ: sulperazone (cefoperazone-sulbactam). Complementary information for better interpretation of the figure above is provided in the text following Figure 4. .

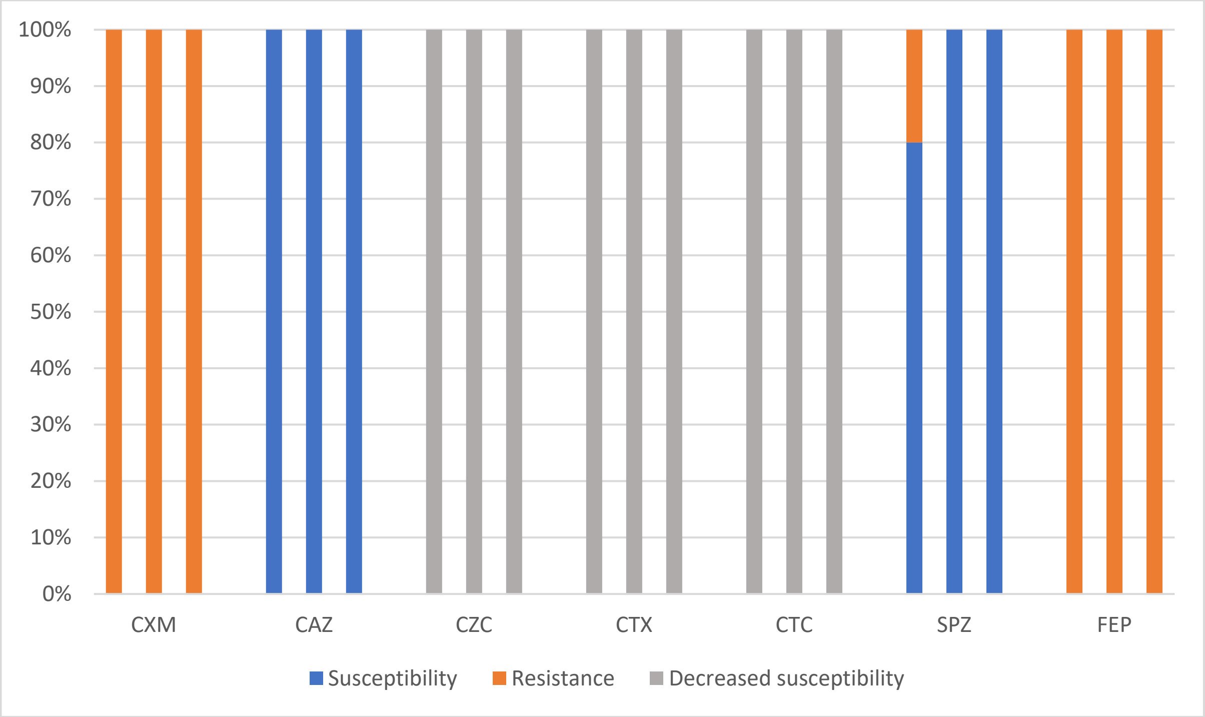

Figure S13. Three-year changes in the antibiotic resistance to cephalosporins of isolated ESBL producing CTX-M variants in *E. coli*. CAZ: ceftazidime, CTX: cefotaxime, CTC: cefotaxime-sulbactam, CXM: cefuroxime, CZC: ceftazidime-sulbactam, FEP: cefepime, SPZ: sulperazone (cefoperazone-sulbactam). Complementary information for better interpretation of the figure above is provided in the text following Figure 4.

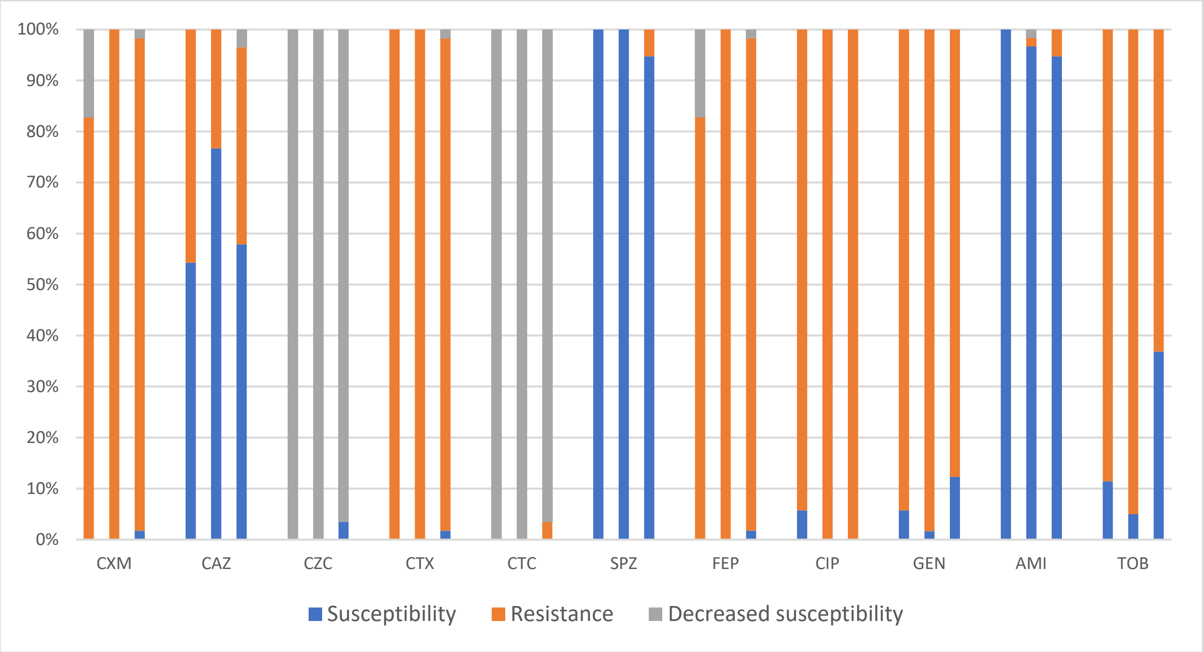

Figure S14. Three-year changes in the antibiotic resistance to cephalosporins, aminoglycosides and fluroquinolones of isolated ESBL producing TEM variants in *P. mirabilis*. AMI: amikacin, CAZ: ceftazidime, CIP: ciprofloxacin, CTX: cefotaxime, CTC: cefotaxime-sulbactam, CXM: cefuroxime, CZC: ceftazidime-sulbactam, FEP: cefepime, GEN: gentamycin, SPZ: sulperazone (cefoperazone-sulbactam), TOB: tobramycin. Complementary information for better interpretation of the figure above is provided in the text following Figure 4.

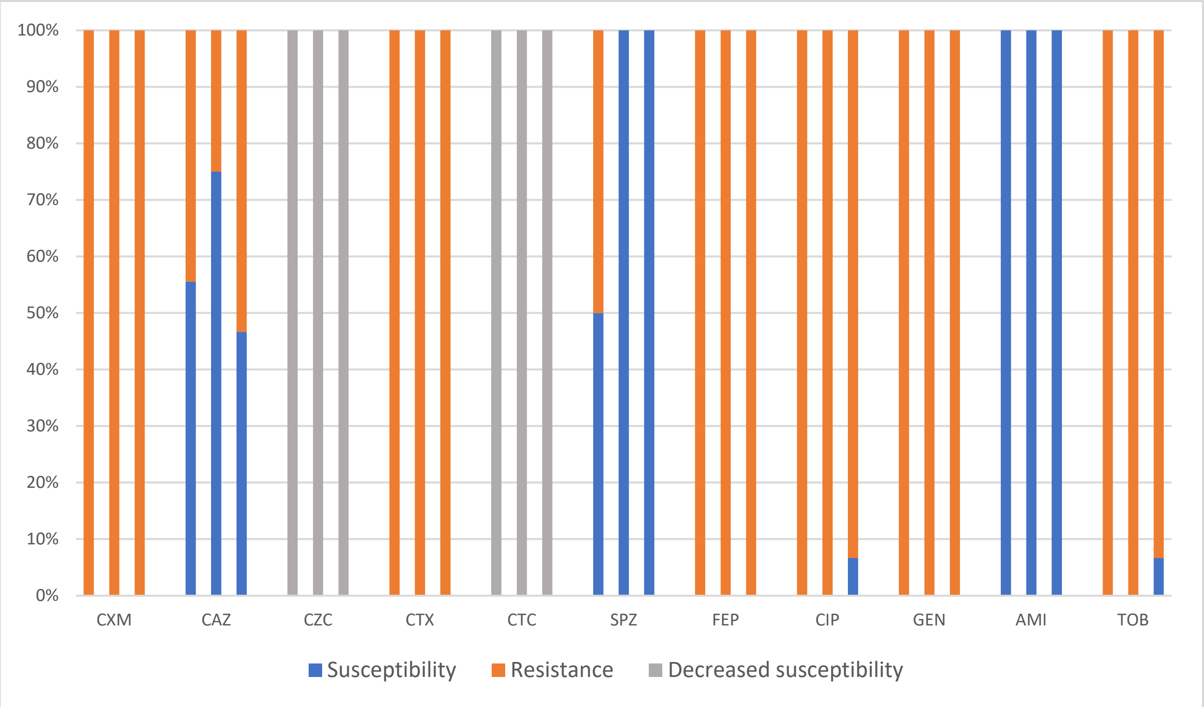

Figure S15. Three-year changes in the antibiotic resistance to cephalosporins, aminoglycosides and fluroquinolones of isolated ESBL producing TEM variants with presence of concomitant AGL ANT (2′)-I phenotype in *P. mirabilis*. AMI: amikacin, CAZ: ceftazidime, CIP: ciprofloxacin, CTX: cefotaxime, CTC: cefotaxime-sulbactam, CXM: cefuroxime, CZC: ceftazidime-sulbactam, FEP: cefepime,

GEN: gentamycin, SPZ: sulperazone (cefoperazone-sulbactam), TOB: tobramycin. Complementary information for better interpretation of the figure above is provided in the text following Figure 4.

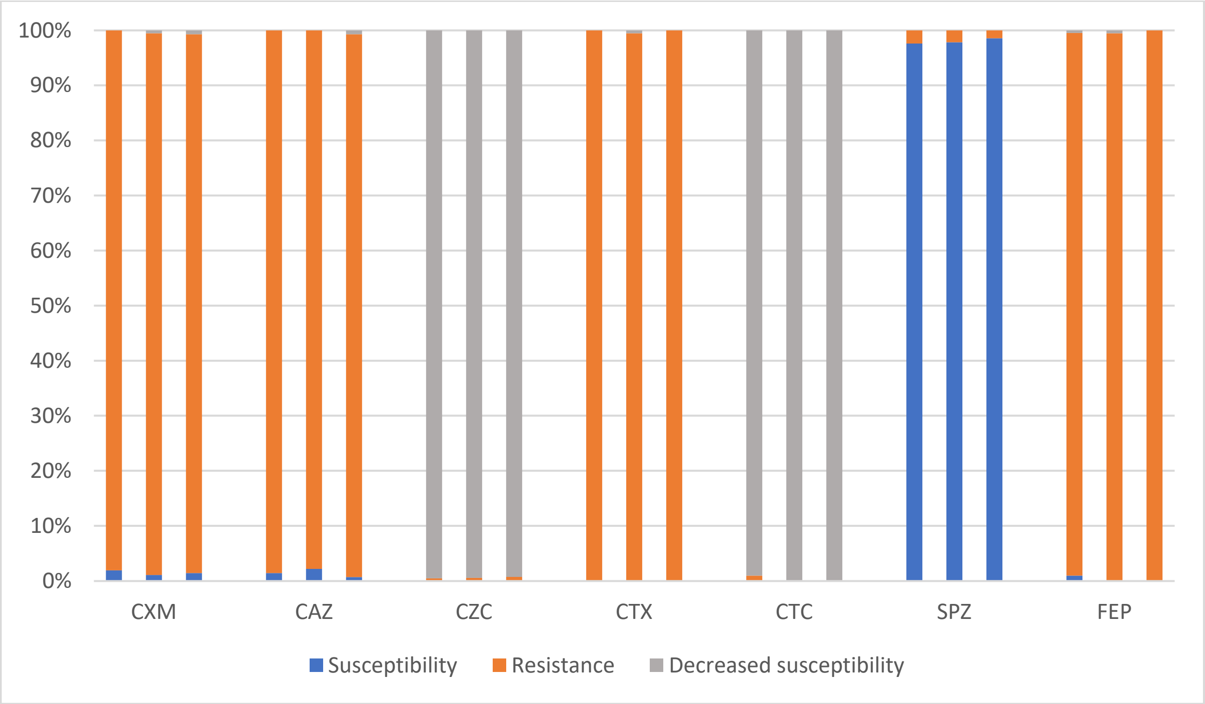

Figure S16. Three-year changes in the antibiotic resistance to cephalosporins of isolated ESBL producing TEM variants in *K. pneumoniae*. CAZ: ceftazidime, CTX: cefotaxime, CTC: cefotaxime-sulbactam, CXM: cefuroxime, CZC: ceftazidime-sulbactam, FEP: cefepime, SPZ: sulperazone (cefoperazone-sulbactam). Complementary information for better interpretation of the figure above is pro-vided in the text following Figure 4.

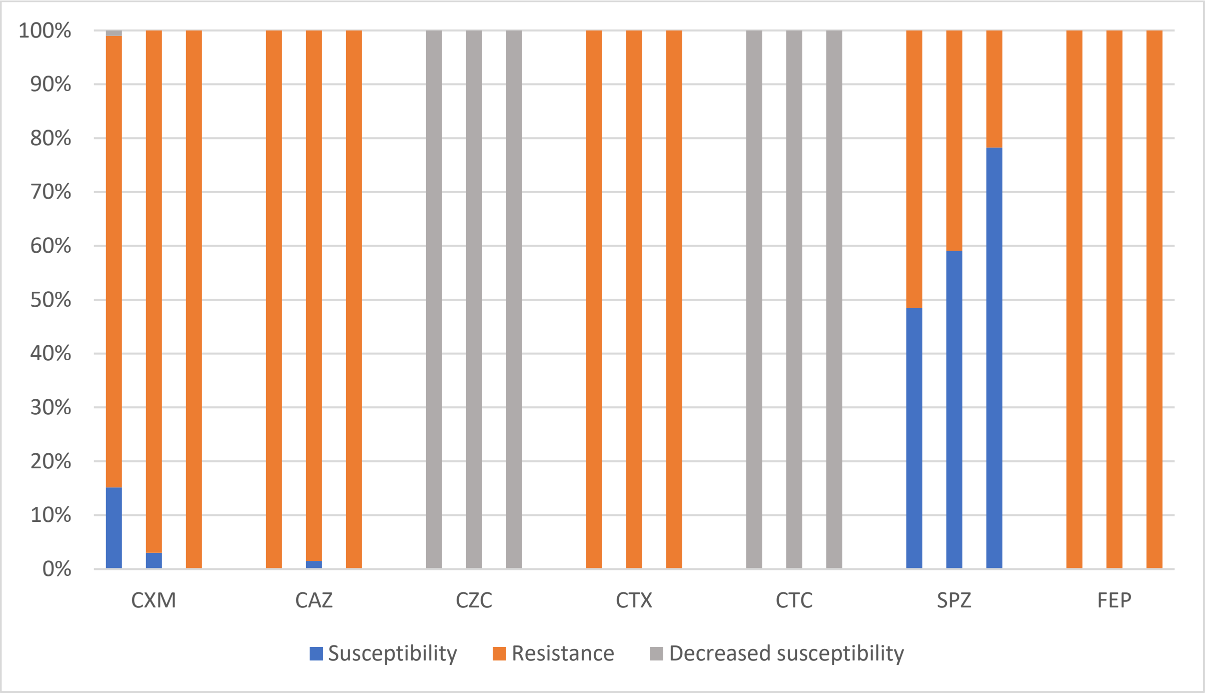

Figure S17. Three-year changes in the antibiotic resistance to cephalosporins of isolated ESBL producing SHV variants in *K. pneumoniae*. CAZ: ceftazidime, CTX: cefotaxime, CTC: cefotaxime-sulbactam, CXM: cefuroxime, CZC: ceftazidime-sulbactam, FEP: cefepime, SPZ: sulperazone (cefoperazone-sulbactam). Complementary information for better interpretation of the figure above is pro-vided in the text following Figure 4.
